# Ferrocenyl-substituted tetrahydrothiophenes via formal [3 + 2]-cycloaddition reactions of ferrocenyl thioketones with donor–acceptor cyclopropanes

**DOI:** 10.3762/bjoc.16.109

**Published:** 2020-06-10

**Authors:** Grzegorz Mlostoń, Mateusz Kowalczyk, André U Augustin, Peter G Jones, Daniel B Werz

**Affiliations:** 1 University of Łódź, Department of Organic & Applied Chemistry, Tamka 12, 91-403 Łódź, Poland; 2 University of Łódź, The Bio-Med-Chem Doctoral School of the University of Lodz and Lodz Instituties of the Polish Academy of Sciences; 3 Technische Universität Braunschweig, Institute of Organic Chemistry, Hagenring 30, 38106 Braunschweig, Germany,; 4 Technische Universität Braunschweig, Institute of Inorganic and Analytical Chemistry, Hagenring 30, 38106 Braunschweig, Germany

**Keywords:** [3 + 2]-cycloaddition reactions, donor–acceptor cyclopropanes, ferrocenyl thioketones, sulfur heterocycles, tetrahydrothiophenes

## Abstract

Ferrocenyl thioketones reacted with donor–acceptor cyclopropanes in dichloromethane at room temperature in the presence of catalytic amounts of Sc(OTf)_3_ yielding tetrahydrothiophene derivatives, products of formal [3 + 2]-cycloaddition reactions, in moderate to high yields. In all studied cases, dimethyl 2-arylcyclopropane dicarboxylates reacted with the corresponding aryl ferrocenyl thioketones in a completely diastereoselective manner to form single products in which (C-2)-Ar and (C-5)-ferrocenyl groups were oriented in a *cis*-fashion. In contrast, the same cyclopropanes underwent reaction with alkyl ferrocenyl thioketones to form nearly equal amounts of both diastereoisomeric tetrahydrothiophenes. A low selectivity was also observed in the reaction of a 2-phthalimide-derived cyclopropane with ferrocenyl phenyl thioketone.

## Introduction

**Scheme 1:**
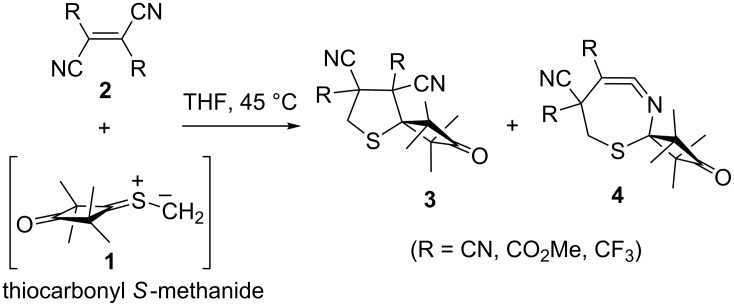
Synthesis of spirotetrahydrothiophenes **3** via non-concerted [3 + 2]-cycloadditions of thiocarbonyl ylide **1** with electron-deficient ethylenes **2**. Cyclic ketene imines **4** are also formed as products of formal [4 + 3]-cycloadditions.

Functionalized tetrahydrothiophenes constitute an important group of five-membered sulfur heterocycles; many of them, both chiral and achiral, with biotin as the best-known representative, form the key motif in numerous compounds of great practical importance [1–2]. The development of chemo- and diastereoselective syntheses for these compounds is thus a challenging problem. An elegant and highly efficient method for the construction of the tetrahydrothiophene ring is based on 1,3-dipolar cycloadditions of in-situ-generated thiocarbonyl *S*-methanides (thiocarbonyl ylides) with electron-deficient ethylenic dipolarophiles. This method was extensively developed by Huisgen and co-workers in the 1980s [3–5]. In the course of these studies, a non-orthodox stepwise mechanism of the 1,3-dipolar cycloaddition was established by experiments performed with the sterically crowded thiocarbonyl *S*-methanide **1**, derived from 2,2,4,4-tetramethyl-3-thioxocyclobutanone and extremely electron-deficient ethylenes **2** such as (*E*)- and (*Z*)-dialkyl dicyanobutenoates (R = CO_2_Me) [6], tetracyanoethylene (R = CN) [7] or (*E*)- and (*Z*)-1,2-bis(trifluoromethyl)ethylene-1,2-dicarbonitrile (R = CF_3_) [8]. Both five-membered spirotetrahydrothiophenes **3** and seven-membered *S,N*-heterocycles (ketene imines) **4** were observed in the course of these reactions (Scheme 1). The latter products were trapped with suitable nucleophiles (R = CO_2_Me) or even isolated and identified by means of spectroscopic methods (R = CF_3_).

**Scheme 2:**
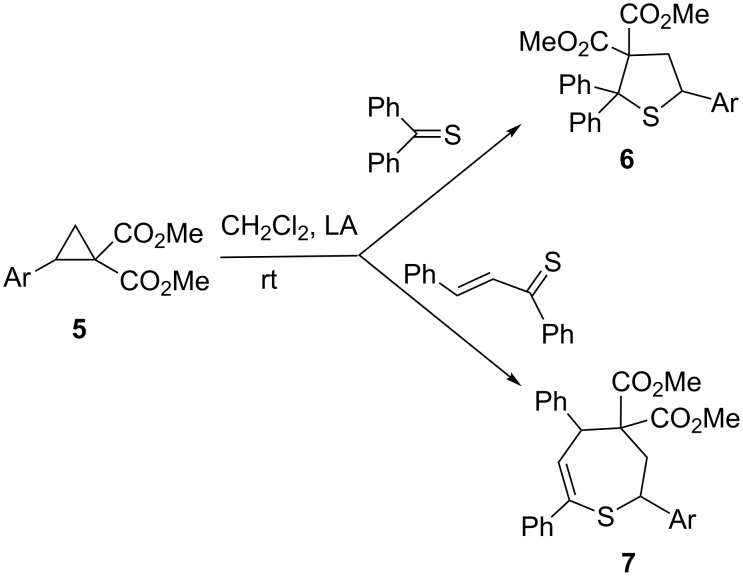
Formal [3 + 2]-cycloadditions of thioketones and [4 + 3]-cycloadditions of thiochalcones with donor–acceptor cyclopropanes **5** leading to tetrahydrothiophenes **6** and tetrahydrothiepines **7**, respectively.

In a recent work, an alternative, efficient and useful method for the synthesis of highly functionalized tetrahydrothiophenes of type **6** was reported [9] (Scheme 2). Under Lewis acid catalysis, formal [3 + 2]-cycloadditions of aromatic and cycloaliphatic thioketones (also thionoesters) with donor–acceptor cyclopropanes **5** (D–A cyclopropanes) were realized.

In contrast, thiochalcones (α,β-unsaturated aromatic thioketones) were shown to react under similar conditions with cyclopropanes **5** yielding exclusively seven-membered tetrahydrothiepines **7** as products of the formal [4 + 3]-cycloaddition [10] (Scheme 2).

In a series of our recent publications, ferrocenyl/aryl and ferrocenyl/alkyl thioketones were demonstrated to be attractive substrates for the preparation of six- and five-membered sulfur heterocycles via [4 + 2]- and [3 + 2]-cycloadditions, respectively [11–15]. Notably, in contrast to aryl/alkyl thioketones (e.g., thioacetophenone), their ferrocenyl analogs of type **8** (e.g., ferrocenyl phenyl thioketone (**8a**), diferrocenyl thioketone (**8b**), and ferrocenyl methyl thioketone (**8c**)) were stable compounds at ambient conditions and could be used with no special precautions. In general, ferrocene has been considered as an ‘exceptional compound’ [16–17] and in our hands ferrocenyl-functionalized sulfur heterocycles, e.g., thiiranes and 1,3-dithiolanes, have found applications for the synthesis of compounds relevant for medicinal [18] and materials chemistry, and electrochemical studies [19].

In continuation of our studies on organic sulfur compounds and the mechanisms of their reactions, the main goal of the present work was the examination of the formal [3 + 2]-cycloaddition reactions of ferrocenyl-substituted thioketones **8** with D–A cyclopropanes **5**, aimed at the synthesis of hitherto unreported, ferrocenyl-substituted tetrahydrothiophene dicarboxylates (thiolanes) of type **9**.

## Results and Discussion

**Scheme 3:**
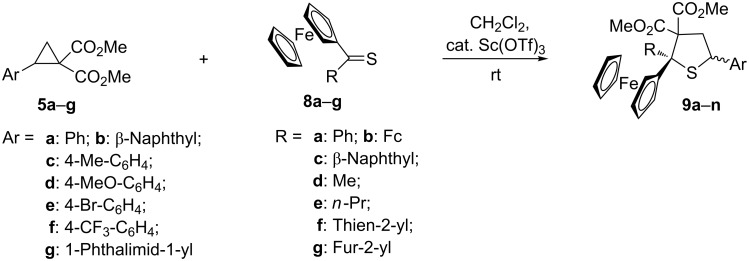
Formal [3 + 2]-cycloadditions of dimethyl 2-substituted cyclopropane-1,1-dicarboxylates **5a**–**g** with ferrocenyl thioketones **8a**–**g**, leading to dimethyl tetrahydrothiophene 3,3-dicarboxylates **9a**–**n** (Table 1).

In analogy to experiments described in our earlier publication [9], the test reaction was performed with dimethyl 2-phenylcyclopropane dicarboxylate (**5a**) and ferrocenyl phenyl thioketone (**8a**) in CH_2_Cl_2_ at room temperature using aluminum chloride (AlCl_3_) as a catalyst. The reaction was monitored by TLC, and was shown to be complete after 1 h. The crude reaction mixture was examined by ^1^H NMR, revealing the formation of a single product with characteristic signals of both CO_2_Me groups located at 3.38 and 3.81 ppm. After chromatographic separation the expected tetrahydrothiophene **9a** was isolated in only 23% yield. As the next model substrate, the sterically crowded diferrocenyl thioketone (**8b**) was tested as a structural analog of thiobenzophenone, which was widely applied in studies involving aromatic thioketones [3–5]. However, in contrast to **8a**, the reaction of **8b** with **5a** was unsuccessful. This observation prompted us to replace AlCl_3_ by scandium triflate (Sc(OTf)_3_), which is also known to be an efficient catalyst in various reactions of D–A cyclopropanes [9–10,20]. This time, the reaction was complete after 1 h and the expected 2,2-diferrocenyl-substituted tetrahydrothiophene **9b** was isolated chromatographically in about 28% yield (Scheme 3, Table 1). This experiment was successfully repeated, again using Sc(OTf)_3_ instead of AlCl_3_, in further experiments of ferrocenyl thioketones **8** with differently substituted cyclopropanes **5**. Again using Sc(OTf)_3_, we repeated the experiment with **8a**, which this time led to the isolation of **9a** in an excellent yield of 98% (Table 1).

**Table 1:** Ferrocenyl-substituted tetrahydrothiophenes **9a**–**n** obtained in reactions of D–A cyclopropanes **5a**–**h** with ferrocenyl thioketones **8a**–**g** catalyzed with Sc(OTf)_3_.

compound **9**	substituent Ar	substituent R	ratio of diastereoisomers	yield of isolated products (%)

**a** **b** **c** **d** **e** **f** **g** **h** **i** **j** **k** **l** **m** **n**	Ph Ph Ph Ph Ph β-naphthyl β-naphthyl 4-Me-C_6_H_4_ 4-MeO-C_6_H_4_ 4-Br-C_6_H_4_ 4-CF_3_-C_6_H_4_ Ph Ph phthalimid-1-yl	Ph Fc^a^ β-naphthyl Me *n*-Pr Ph β-naphthyl Ph Ph Ph Ph thien-2-yl fur-2-yl Ph	100: 0 – 100:0 55:45 52:48 100:0 100:0 100:0 100:0 100:0 100:0 100:0 60:40 60:40	98 28 65 98 97 30 31 85 79 93 95 58 96 34

^a^Fc = ferrocenyl.

**Figure 1:**
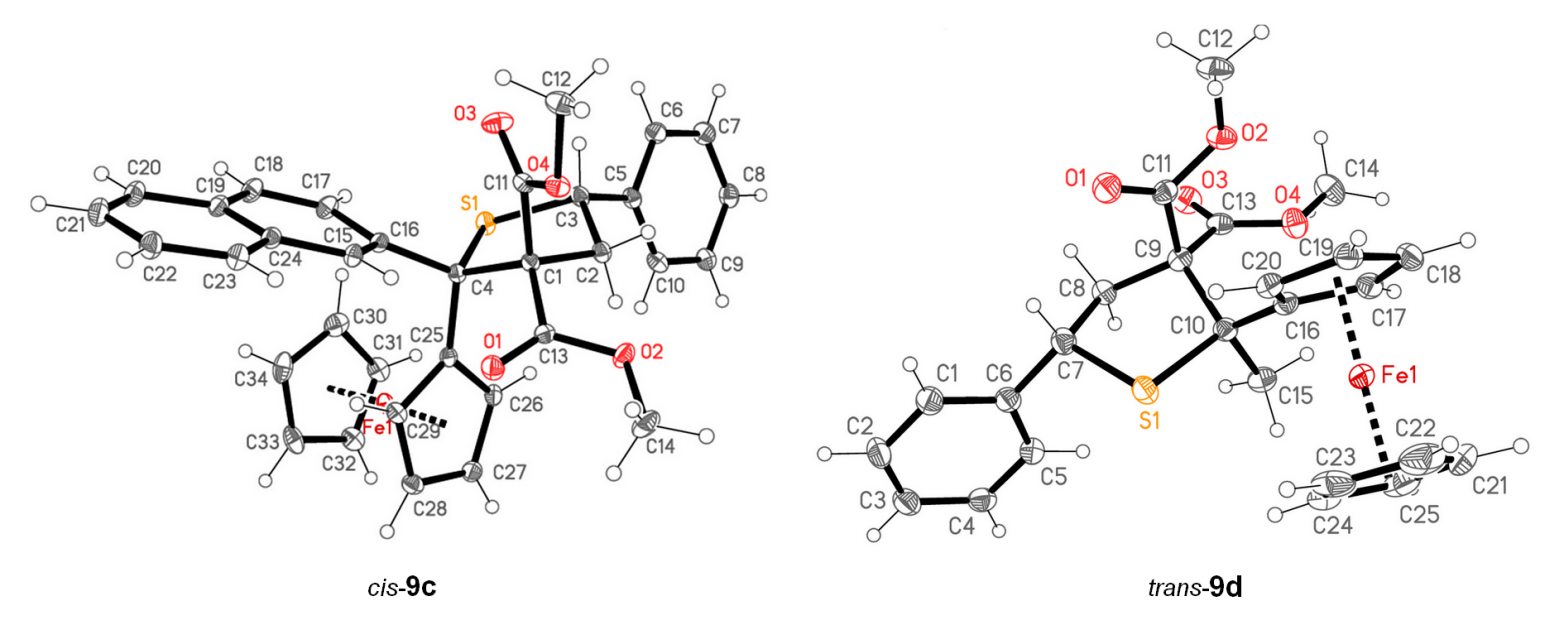
Thermal ellipsoid plots of the molecular structures of *cis*-**9c** and *trans*-**9d** drawn using 50% probability displacement ellipsoids. The terminology *cis* and *trans* referred to the relative orientation of Ph and Fc groups.

In analogy to **8a**, the similarly substituted ferrocenyl (β-naphthyl) thioketone (**8c**) reacted with **5a** in a diastereoselective manner yielding the expected product **9c** in good yield (65%) as the sole isolated product. Notably, in all reactions performed with aryl-substituted cyclopropanes **5a**–**f** and with thioketones **8a**,**c**,**f**, the desired tetrahydrothiophenes **9a**,**c**,**f**–**l** were formed with complete diastereoselectivity, leading to a single isomer. In order to establish the structure of the isomers, a single crystal obtained for compound **9c** was studied by X-ray diffraction analysis which showed, that the Ph(C-2) group and Fc(C-5) substituent were mutually *cis*-oriented (Figure 1). Tentatively, the same configuration was also attributed to all tetrahydrothiophenes **9a**,**f**–**l** that were formed as single isomers (Table 1).

However, the diastereoselectivity changed in reactions that were conducted with alkyl ferrocenyl thioketones **8d**–**e** with **5a**. Thus, the reaction with **8d** led to a 55:45 mixture of two isomeric products in nearly quantitative yield (98%). Subsequently, they were carefully separated by preparative thin layer chromatography (PTC) on silica using a mixture of petroleum ether and ethyl acetate as an eluent. The less polar fraction formed the major product and the slightly more polar one was isolated and identified as the minor isomer of **9d**. In the course of crystallization from hexane the less polar fraction gave single crystals suitable for the X-ray diffraction analysis, which unambiguously confirmed that in this molecule the Ph(C-5) and Fc(C-2) groups were *trans*-oriented and for that reason, this isomer was described as *trans*-**9d** (Figure 1).

Analogously, the reaction of ferrocenyl *n*-propyl thioketone (**8e**) with **5a** led to a 52:48 mixture of *trans*- and *cis*-isomers of **9e**, which were isolated in a total yield of 97% and identified without further separation. Moreover, a mixture of nearly equal amounts of isomeric *trans*-**9m** and *cis*-**9m** was also observed in the reaction of **5a** with ferrocenyl fur-2-yl thioketone (**8g**). The reaction of the phthalimide-derived cyclopropane **5g** with thioketone **8a** led to a 4:1 mixture of both isomers *cis*- and *trans*-**9n**. Based on these observations it was difficult to explain the complete diastereoselectivity of tetrahydrothiophene formation observed in the reactions of aryl ferrocenyl-substituted thioketones **8a**,**c**,**f** with cyclopropanes **5a**–**f** bearing aryl groups. Tentatively, a repulsive interaction of aryl groups rather than steric hindrance of the bulky ferrocenyl unit could be postulated. Remarkably, ferrocenyl fur-2-yl thioketone (**8g**) was an exception and delivered a 60:40 mixture of *trans*- and *cis*-**9m**.

**Scheme 4:**
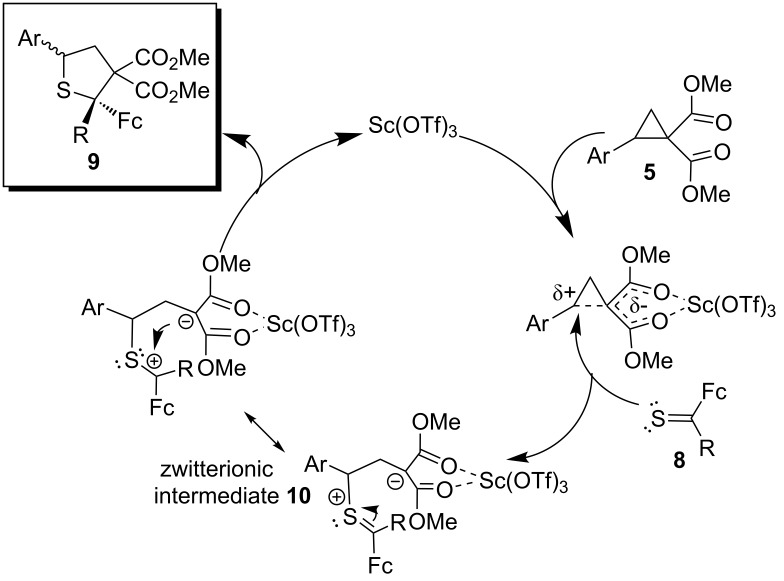
Plausible mechanism for the formal [3 + 2]-cycloadditions of ferrocenyl thioketones **8** with D–A cyclopropanes **5**.

The mechanistic interpretation of the efficient, formal [3 + 2]-cycloadditions of D–A cyclopropanes **5** with ferrocenyl thioketones **8** in the presence of a Lewis acid was based on the assumption that the coordination of the catalyst by two ester groups activated the cyclopropane ring and allowed a nucleophilic attack of the C=S group on the benzylic position of the cyclopropane derivative (Scheme 4).

The subsequent ring-closure of the zwitterionic intermediate **10** led to the formation of the tetrahydrothiophene derivative **9**. This process formally resembled the [3 + 2]-cycloadditions of thiocarbonyl *S*-methanides with an activated C–C double bond, which also led to tetrahydrothiophenes [6–8]. Nevertheless, the key step involved the formation of the reactive, zwitterionic intermediate **10**. It seemed that repulsive interactions of the aryl groups Ar (from cyclopropane **5**) and R (from thioketone **8**) controlled the diastereoselective ring-closure to the five-membered ring leading in these cases to the formation of *cis*-**9** (Ar to Fc) as a single isomer. A similar reaction pathway with a zwitterionic intermediate analogous to **10**, generated in the presence of a Lewis acid, was proposed for the reaction of cycloaliphatic 3-thioxo-2,2,4,4-tetramethylcyclobutanone with D–A cyclopropanes [21].

## Conclusion

The present study showed once more that ferrocenyl/aryl and ferrocenyl/alkyl thioketones **8** are versatile and useful building blocks for a simple and efficient preparation of ferrocenyl-functionalized five-membered sulfur heterocycles. They were shown to react easily with donor–acceptor (D–A) cyclopropanes in the presence of scandium triflate, Sc(OTf)_3_ as a catalyst, yielding highly functionalized tetrahydrothiophene derivatives of type **9**. These formal [3 + 2]-cycloaddition reactions occurred via a nucleophilic attack of the sulfur atom on the activated cyclopropane ring at the most reactive benzylic position. The formation of the five-membered ring occurred regioselectively and the expected tetrahydrothiophene-3,3-carboxylates were the products. The studied reactions displayed an interesting stereoselectivity and, in the case of 2,5-diaryl-substituted products **9**, both aryl groups were exclusively located at the opposite sides of the ring plane. The described reactions supplement the recently reported synthetic applications of alkyl/ferrocenyl thioketones as attractive substrates for the synthesis of chiral ferrocene derivatives [22] and ferrocenyl-substituted platinathiiranes [23].

It should be also emphasized that the present study also demonstrated the growing potential of donor–acceptor cyclopropanes [24–28] as unique building blocks for current organic synthesis and especially for the efficient and highly stereoselective preparation of the relevant five-membered sulfur heterocycles derived from tetrahydrothiophene.

## Experimental

**General information:** Solvents and chemicals were purchased and used as received without further purification. Products were purified by standard column chromatography on silica gel. Yields refer to analytically pure samples. NMR spectra were recorded with a Bruker Avance III 600 MHz instrument (^1^H NMR: 600 MHz; ^13^C NMR: 151 MHz). Chemical shifts are reported relative to solvent residual peaks (^1^H NMR: δ = 7.26 ppm [CHCl_3_]; ^13^C NMR: δ = 77.0 ppm [CDCl_3_]). IR spectra were recorded with a Cary 630 FTIR (Agilent Technologies) spectrometer (as film). Melting points were determined in capillaries with a Melt Temp II apparatus.

**Starting materials:** D–A cyclopropanes **5a**–**g** were obtained following the reported procedure [28]. Ferrocenyl thioketones **8a**–**g** were obtained by thionation of corresponding ferrocenyl ketones [29] by treatment with Lawesson’s reagent [30]. Ferrocenyl β-naphthyl thioketone (**8b**) obtained from ferrocenyl(β-naphthyl) ketone [31] is reported for the first time (see Supporting Information File 1).

**General procedure:** A solution of 0.3 mmol of the corresponding cyclopropane **5** in 5 mL of dichloromethane was stirred for 5 min. Then, 0.5 mmol of the corresponding ferrocenyl thioketone **8** and a catalytic amount (ca. 5 mg) of Sc(OTf)_3_ was added to the stirred solution. The mixture was stirred at room temperature for 1 h. The progress of the reaction was monitored by TLC. The solvent was evaporated in vacuo and the crude mixture was purified by flash chromatography using dichloromethane as the eluent. Analytically pure samples of tetrahydrothiophenes **9** were obtained by crystallization from petroleum ether or hexane with a small amount of dichloromethane.

The diastereoselectivity of the studied reactions was determined by integration of the crude ^1^H NMR. Preliminary purification of crude mixtures by a short-column chromatography was necessary to remove traces of iron particles formed as a side product after partial decomposition of ferrocenyl containing substrates and/or products formed under reaction conditions.

**Dimethyl 2-ferrocenyl-2,5-diphenyltetrahydrothiophene-3,3-dicarboxylate (*cis*-9a):** Yield: 159 mg (98%); red crystals; mp 192–193°C; ^1^H NMR δ 2.65 (dd, *J*_H,H_ = 13.9 Hz, *J*_H,H_ = 4.3 Hz, 1H, *H*C(4)), 3.44 (s, 3H, OCH_3_), 3.46 (s, 3H, OCH_3_), 3.56 (dd, *J*_H,H_ = 13.9 Hz, *J*_H,H_ = 12.7 Hz, 1H, *H*C(4)), 3.51–3.60 (m, 1H, *H*C(Fc)), 4.00–4.02 (m, 1*H*C(Fc)), 4.07 (s, 5*H*C(Fc)), 4.27–4.29 (m, 1*H*C(Fc)), 4.68–4.70 (m, 1 *H*C(Fc)), 4.81 (dd, *J*_H,H_ = 12.6 Hz, *J*_H,H_ = 4.3 Hz, *H*C(5)), 7.31–7.39 (m, 2 arom. *H*C), 7.40–7.46 (m, 4 arom. *H*C), 7.62–7.65 (m, 2 arom. *H*C), 8.21–8.24 (m, 2 arom. *H*C); ^13^C NMR δ (C(4)-not found), 48.1, 48.4 (2O*C*H_3_), 52.4 (C(5)), 67.8 (C(2)), 71.1 (C(3)), 68.7, 69.2, 69.9, 71.0, 73.7 (for 9 H*C*(Fc)), 97.0 (*C*(Fc)), 126.6, 127.1, 127.9, 128.0, 128.8, 129.0 (for 10 arom. H*C*), 138.9, 144.2 (2 arom. *C*), 169.0, 170.3 (2 *C*=O); IR (cm^−1^) ν: 1737 brs (2C=O), 1492 m, 1444 m, 1429 m, 1258 m, 1239 s, 1073 m, 814 m, 760 m, 697 vs, 497 vs; Anal. calcd for C_30_H_28_FeO_4_S (540.45): C, 66.67; H, 5.22; S, 5.93; found: C, 66.58; H, 5.24; S, 5.99.

**Dimethyl 2,2-diferrocenyl-5-phenyl tetrahydrothiophene-3,3-dicarboxylate (9b):** Yield: 54 mg (28%); red crystals; mp 170 °C (dec.); ^1^H NMR δ 2.91 (dd, *J*_H,H_ = 14.0 Hz, *J*_H,H_ = 6.2 Hz, 1H, *H*C(4)), 3.36 (s, 3H, OCH_3_), 3.41 (dd, *J*_H,H_ = 15.8 Hz, *J*_H,H_ = 12.4 Hz, 1H, *H*C(4)), 3.65 (s, 3H, OCH_3_), 4.05–4.07 (m, 1H, *H*C(Fc)), 4.07–4.09 (m, 1H, *H*C(Fc)), 4.16–4.18 (m, 1H, *H*C(Fc)), 4.23–4.27 (m, 7H, 7*H*C(Fc)), 4.31 (s, 5H, *H*C(Fc)), 4.48–4.50 (m, 1H, *H*C(Fc)), 4.56–4.58 (m, 1H, *H*C(Fc)), 4.68–4.70 (m, 1H, *H*C(Fc)), 5.51 (dd, *J*_H,H_ = 11.2 Hz, *J*_H,H_ = 6.3 Hz, 1H, *H*C(5)), 7.34–7.37 (m, 1arom. *H*C), 7.44–7.48 (m, 2arom. *H*C), 7.78 (m, 2arom. *H*C); ^13^C NMR δ 47.9 (C(4)), 49.5 (C(5)), 51.9, 52.6 (2O*C*H_3_), 65.8, 66.2, 66.9, 67.6, 67.7, 69.6, 69.7, 70.4, 73.1 (for 18 H*C*(Fc), 73.4, 94.3 (C(2) and C(3), 100.0 (2*C*(Fc)), 127.4, 128.2, 128.6 (5 arom. H*C*), 141.2 (arom. *C*), 169.0, 169.1 (2 *C*=O); IR (cm^−1^) ν: 1727 brs (2C=O), 1431 m, 1259 s, 1164 s, 1107 m, 1000 m, 818 s, 760 m, 696 s, 479 vs; anal. calcd for C_34_H_32_Fe_2_O_4_S (648.37): C, 62.98; H, 4.97; S, 4.94; found: C, 62.68; H, 4.93; S, 4.88.

**Dimethyl 2-ferrocenyl-5-phenyl-2-(naphth-2-yl)tetrahydrothiophene-3,3-di-carboxylate (*cis*-9c):** Yield: 115 mg (65%); yellow crystals; mp 210–211 °C; single crystals were obtained from hexane solution by slow evaporation at rt; ^1^H NMR δ 2.69 (dd, *J*_H,H_ = 13.8 Hz, *J*_H,H_ = 4.3 Hz, 1H, *H*C(4)), 3.41 (s, 3H, OCH_3_), 3.47 (s, 3H, OCH_3_), 3.57 (s, 1H, *H*C(Fc)), 3.62 (t, *J*_H,H_ = 13.1 Hz, 1H, CH), 4.01 (s, 1H, *H*C(Fc)), 4.09 (s, 5 *H*C(Fc)), 4.31 (s, 1H, *H*C(Fc)), 4.77 (s, 1H, *H*C(Fc)), 4.86 (dd, *J*_H,H_ = 12.6 Hz, *J*_H,H_ = 4.3 Hz, 1H, *H*C(5)), 7.36–7.40 (m, 1 arom., *H*C), 7.44–7.48 (m, 2 arom. *H*C), 7.51–7.54 (m, 2 arom. *H*C), 7.66 (m, 2 arom. *H*C), 7.87 (d, *J*_H,H_ = 8.6 Hz, 1 arom., *H*C), 7.88–7.92 (m, 1 arom., *H*C), 7.95–7.99 (m, 1 arom., *H*C), 8.36 (d, *J*_H,H_ = 8.6 Hz, 1arom., *H*C), 8.74 (s, 1 arom., *H*C); ^13^C NMR δ 48.1 (C(5)), 48.5 (C(4)), 52.5, 52.6 (2O*C*H_3_), 67.8, 68.7, 69.2, 69.9, 71.2 (for 9 H*C*(Fc)), 71.0, 73.5 (C(2) and C(3)) 97.4 (2 *C*(Fc)), 125.8, 125.9, 126.1, 127.2, 127.3, 127.9, 128.0, 128.1, 128.6, 128.8 (10 arom. H*C*), 132.1, 132.8, 138.8, 141.8 (4 arom. *C*), 168.9, 170.3 (2 *C*=O); IR (cm^−1^) ν: 1738 brs (2C=O), 1429 m, 1239 s, 1215 s, 1170 m, 1053 m, 810 s, 758 m, 704 s, 480 vs; anal. calcd for C_34_H_30_FeO_4_S (590.51): C, 69.15; H, 5.12; S, 5.43; found: C, 67.16; H, 5.01; S, 5.47.

**Dimethyl 2-ferrocenyl-2-phenyl-5-methyltetrahydrothiophene-3,3-dicarboxylate (9d)**. Obtained as a 55:45 mixture of isomers. The *trans*- (major) and *cis*- (minor) isomers (Ph to Fc) were separated by PLC (silica, PE/ethyl acetate). Yields: *cis-*isomer, yellow crystals, 66 mg (more polar fraction, 44%); mp 148–150 °C, *trans*-isomer, yellow crystals, 74 mg (less polar fraction, 54%); mp 126–128 °C; single crystals of *trans*-**9d** were obtained from hexane/CH_2_Cl_2_ solution by slow evaporation at rt; ^1^H NMR (*cis*-**9d**) δ 2.31 (s, 3H, CH_3_); 2.54 (dd, *J*_H,H_ = 13.8 Hz, *J*_H,H_ = 5.3 Hz, 1H, *H*C(4)); 3.22 (dd, *J*_H,H_ = 13.8 Hz, *J*_H,H_ = 12.3 Hz, 1H, *H*C(4)); 3.46 (s, 3H, OCH_3_); 3.80 (s, 3H, OCH_3_); 4.09–4.10 (m, 1H, *H*C(Fc)); 4.12–4.13 (m, 1H, *H*C(Fc)); 4.22 (s, 5H, HCH(Fc)); 4.23–4.25 (m, 1H, *H*C(Fc)); 4.55–4.56 (*m*, 1H, HC(Fc)); 4.78 (dd, *J*_H,H_ = 12.3 Hz, *J*_H,H_ = 5.3 Hz, 1H, *H*C(5)); 7.30–7.34 (m, 1 arom. *H*C); 7.39–7.43 (m, 2 arom. *H*C); 7.54–7.58 (m, 2 arom. *H*C); ^13^C NMR (*cis*-**9d**) δ 25.9 (*C*H_3_); 43.9 (C(4)); 47.9 (C(5)); 52.0, 52.6 (2O*C*H_3_); 60.0 (C(2)); 68.1, 68.2, 68.8, 69.1, 70.7 (for 9 H*C*(Fc)); 70.6 (C(3)); 96.5 (*C*(Fc)); 127.5, 127.6, 128.7 (5 arom. H*C*); 140.0 (arom. *C*); 168.5, 169.7 (2*C*=O); IR (cm^−1^) ν: 1731 brvs (2C=O); 1494 m, 1453 m, 1436 m, 1248 vs, 1207 m, 1157 vs, 1105 m, 1038 s, 829 m, 766 s, 702 vs; anal. calcd for C_25_H_26_FeO_4_S (478.38): C, 62.77; H, 5.48; S, 6.70; found: C, 62.69; H, 5.52; S, 6.63.

^1^H NMR (*trans*-**9d**) δ 2.28 (s, 3H, CH_3_); 2.79 (dd, *J*_H,H_ = 14.1 Hz, *J*_H,H_ = 10.7 Hz, 1H, *H*C(4)); 3.12 (dd, *J*_H,H_ = 14.1 Hz, *J*_H,H_ = 7.1 Hz, 1H, *H*C(4)); 3.47 (s, 3H, OCH_3_); 3.66 (s, 3H, OCH_3_); 4.19–4.21 (m, 2H, *H*C(Fc)); 4.23 (s, 5H, 5*H*C(Fc)); 4.40–4.42 (m, 1H, *H*C(Fc)); 4.56–4.57 (m, 1H, *H*C(Fc)); 5.28 (dd, *J*_H,H_ = 10.7 Hz, *J*_H,H_ = 7.1 Hz, 1H, *H*C(5)); 7.30–7.32 (m, 1 arom. *H*C); 7.39–7.42 (m, 2 arom. *H*C); 7.57–7.59 (m, 2 arom. *H*C); ^13^C NMR (*trans-***9d**) δ 31.3 (CH_3_); 47.1 (C(4)); 48.6 (C(5)); 52.2, 52.3 (2O*C*H_3_); 60.1 (C(2)); 67.6 (C(3)); 68.5, 68.8, 69.1, 69.3, 71.1 (for 9H*C*(Fc)); 89.9 (*C*(Fc)); 127.3, 127.9, 128.6 (5 arom. H*C*); 142.6 (arom. *C*); 168.9, 169.5 (2*C*=O); IR (cm^−1^) ν: 1737 vs, 1720 vs (2C=O); 1492 m, 1453 m, 1427 m, 1258 vs, 1220 s, 1204 m, 1106 m, 1105 m, 1023 m, 993 m, 829 m, 766 s, 703 vs; anal. calcd for C_25_H_26_FeO_4_S (478.38): C, 62.77; H, 5.48; S, 6.70; found: C, 62.70; H, 5.46; S, 6.59.

**Dimethyl 2-ferrocenyl-5-phenyl-2-(thien-2-yl)tetrahydrothiophene-3,3-dicarboxy-late (*trans*-9l):** Yield: 95 mg (58%); yellow crystals; mp 210 °C (dec.); ^1^H NMR δ 2.65 (dd, *J*_H,H_ = 14.0 Hz, *J*_H,H_ = 4.5 Hz, 1H, *H*C(4)), 3.43 (pseudo-t, *J*_H,H_ = 13.9 Hz, 1H, *H*C(4)), 3.48 (s, 3H, OCH_3_), 3.52 (s, 3H, OCH_3_), 4.10 (s, 5H, 5*H*C(Fc)), 4.14 (s, 2H, 2*H*C(Fc)), 4.30 (s, 1H, *H*C(Fc)), 4.68 (s, 1H, HC(Fc)), 5.02 (dd, *J*_H,H_ = 13.6 Hz, *J*_H,H_ = 4.5 Hz, 1H, *H*C(5)), 7.05–7.07 (m, 1 arom. *H*C), 7.21–7.23 (m, 1 arom. *H*C), 7.34–7.38 (m, 1 arom. *H*C), 7.42–7.45 (m, 2 arom., *H*C), 7.55–7.57 (m, 1 arom. *H*C), 7.60–7.63 (m, 2 arom. *H*C); ^13^C NMR δ 47.3 (C(4)), 49.0 (C(5)), 52.2, 52,5 (2O*C*H_3_), 67.9, 69.0, 69.4, 70.4, 70.7 (for 9 H*C*(Fc)), 66.3, 74.2 (2 arom. *C*), 94.6 (1 *C*(Fc)), 123.4, 125,8, 126.8, 127.9, 128.0, 128.8 (for 8 arom. H*C*), 138.7, 150.3 (2 arom. *C*), 168.4, 169.5 (2*C*=O); IR (cm^−1^) ν: 1733 brs (2C=O), 1427 m, 1235 s, 1146 s, 1045 m, 1032 m, 818 m, 766 s, 691 vs, 506 m, 488 s; HRMS–EI (*m*/*z*): [M]^+^ calcd. for [C_28_H_26_FeO_4_S_2_]^+^, 546.0621; found: 546.0629.

## Supporting Information

CCDC-1992864 and CCDC-1992865 contain the supplementary crystallographic data for this paper. These data can be obtained free of charge from the Cambridge Crystallographic Data Centre via http://www.ccdc.cam.ac.uk/structures.

File 1 Experimental data for selected compounds **9**, details of the crystal structure determination, and the original ^1^H and ^13^C NMR spectra for all products.
