# Supplementary material for: Ferrocenyl-substituted tetrahydrothiophenes via formal [3 + 2]-cycloaddition reactions of ferrocenyl thioketones with donor–acceptor cyclopropanes
Source: Beilstein J Org Chem. 2020 Jun 10;16:1288–95. doi: 10.3762/bjoc.16.109 (PMC7296194; doi:10.3762/bjoc.16.109)
Supplement: File 1 — Experimental data for selected compounds 9, details of the crystal structure determination, and the original 1H and 13C NMR spectra for all products. [file Beilstein_J_Org_Chem-16-1288-s001.pdf]

BEILSTEIN JOURNAL OF ORGANIC CHEMISTRY

Supporting Information

for

Ferrocenyl-substituted tetrahydrothiophenes via formal [3 + 2]-cycloaddition reactions of ferrocenyl thioketones with donor–acceptor cyclopropanes

Grzegorz Mlostoń, Mateusz Kowalczyk, André U. Augustin, Peter G. Jones and Daniel B. Werz

Beilstein J. Org. Chem. **2020,** *16,* 1288–1295. doi:10.3762/bjoc.16.109

Experimental data for selected compounds 9, details of the crystal structure determination, and the original ^{1}H and ^{13}C NMR spectra for all products

License and Terms: This is a supporting information file under the terms of the Creative Commons Attribution License (http://creativecommons.org/licenses/by/4.0). Please note that the reuse, redistribution and reproduction in particular requires that the authors and source are credited.
The license is subject to the *Beilstein Journal of Organic Chemistry* terms and conditions: (https://www.beilstein-journals.org/bjoc)

1. Experimental

1. 1. Experimental data for thioketone 8c

Ferrocenyl (2-naphthyl)methanthione (8c): Yield: 1.09 g (92%). Deep blue crystals; mp 127-128°C; ^{1}H-NMR (600 MHz, $CDCl_3$): δ 4.19 (s, 5*H*C(Fc)); 4.85 (s, 2*H*C(Fc)); 5.07 (s, 2*H*C(Fc)); 7.36-7.43 (m, 3 arom. *H*C); 7.47-7.54 (m, 2 arom. *H*C); 7.66-7.73 (m, 2 arom. *H*C); ^{13}C-NMR (151 MHz, $CDCl_3$): δ 72.3, 72.9, 74.9 (for 9H*C*(Fc)), 89.6 (*C*(Fc)), 125.6, 126.0, 126.7, 127.3, 127.4, 127.7, 129.1 (7 arom. H*C*), 132.3, 134.2, 146.2 (3 arom. *C*), 238.1 (*C*=S); Anal. calcd for $C_{21}H_{16}SFe$ (356.26): C 70.80; H 4.53; S 9.00; found C 70.67; H 4.69; S 9.02.

1. 2. Experimental data for tetrahydrothiophenes 9e–k,m–n

Dimethyl 2-ferrocenyl-5-phenyl-2-(*n*-propyl)tetrahydrothiophene-3,3-dicarboxylate (9e): Yield: 147 mg (97%); isolated chromatographically as a 52:48 mixture of isomers. The major component was isolated as a less polar fraction by repeated preparative thin layer chromatography on silica. Yellow crystals; mp 109-110°C; ^{1}H-NMR (600 MHz, $CDCl_3$) (major isomer): *δ* 1.12 (t, *J* = 7.2 Hz, 3H, CH_3(*n*-Pr)), 1.29 (t, *J* = 7.1 Hz, 2H, CH_2(*n*-Pr)), 2.75 (dd, *J* = 13.9, *J* = 11.1 Hz, 1H, *H*C(4)) 3.00 (dd, *J* = 13.9, *J* = 6.8 Hz, 1H, *H*C(4)) 3.41 (s, 3H, OCH_3), 3.67 (s, 3H, OCH_3), 4.15 (q, *J* = 14.3, *J* = 7.1 Hz, CH_2(*n*-Pr)), 4.18 (s, 6*H*C(Fc)), 4.20 (s, 1*H*C(Fc)), 4.38 (*s*, 1*H*C(Fc)), 4.57 (*s*, 1*H*C(Fc)), 5.43 (*dd*, *J* = 11.0, 6.8 Hz, 1H, *H*C(5)), 7.36-7.40 (*m*, 2 arom. *H*C), 7.53-7.56 (*m*, 2 arom. *H*C); ^{13}C-NMR (151 MHz, $CDCl_3$) (major isomer): *δ* 15.1 (*C*H_3), 20.2 (CH_2*C*H_2CH_3), 44.2 (*C*$H_2CH_2CH_3$), 47.3 (C(4)) 48.9 (H*C*(5)), 52.2, 52.5 (2O*C*H_3), 65.5, 71.7 (C(2) and C(3)), 67.1, 68.1, 68.2, 69.3, 69.4 (for 9H*C*(Fc)), 91.5 (*C*(Fc)), 127.3, 127.7, 128.7 (for 5 arom. H*C*), 142.1 (1 arom. *C*), 169.2, 170.1 (2*C*=O); IR: ν 1731*brs* (2C=O), 1449*m*, 1431*m*, 1241*s*, 1209*s*, 1105*m*, 1051*m*, 818*s*, 762*m*, 697*vs*, 482*vs* cm^{-1}; HRMS–EI (*m*/*z*): $[M]^+$ calcd. for $[C_{27}H_{30}O_4SFe]^+$, 506.1214; found, 506.1211.

Dimethyl 2-ferrocenyl-5-(2-naphthyl)-2-phenyltetrahydrothiophene-3,3-dicarboxylate (*cis*-9f): Yellow crystals, 22 mg (30%); mp 178-180°C. ^{1}H-NMR (600 MHz, $CDCl_3$): *δ* 2.73 (dd, *J* = 13.9, *J* = 4.3 Hz, 1H, *H*C(4)), 3.46 (s, 3H, OCH_3), 3.49 (s, 3H, OCH_3), 3.59-3.61 (m, 1*H*C(Fc)), 3.69 (dd, *J* = 13.7, *J* = 12.9 Hz, 1H, *H*C(4)), 4.02-4.04 (m, 1*H*C(Fc)), 4.07 (s, 5*H*C(Fc)), 4.30-4.31 (m, 1*H*C(Fc)), 4.73-4.75 (m, 1*H*C(Fc)), 5.00 (dd, *J* = 12.6, *J* = 4.3 Hz, 1H, *H*C), 7.33-7.39 (m, 1 arom. *H*C), 7.41-7.46 (m, 2 arom. *H*C), 7.51-7.56 (m, 2 arom. *H*C), 7.81-

7.86 (m, 1 arom. *H*C), 7.89-7.91 (m, 2 arom. *H*C), 7.94-7.96 (m, 1 arom. *H*C), 8.00 (s, 1 arom. *H*C), 8.24-8.27 (m, 2 arom. *H*C); ^{13}C-NMR (151 MHz, $CDCl_3$): δ 48.0 (C(4)), 48.2 (C(5)), 52.5, 52.6 (OCH_3), 67.8, 68.7, 69.1, 69.9, 71,1 (for 9H*C*(Fc)), 73.7 (for C(2) and C(3)), 96.9 (*C*(Fc)), 125.7, 126.1, 126.3, 126.6, 126.9, 127.1, 127.7, 127.8, 128.5, 128.8 (for 12 arom. H*C*), 133.1, 133.4, 136.1, 144.2 (4 arom. *C*), 168.9, 170.3 (2*C*=O); IR: ν 1744*vs* (2C=O), 1444*m*, 1427*m*, 1258*m*, 1239*vs*, 1187*m*, 1168*m*, 1051*m*, 814*m*, 723*m*, 698*m*, 480*vs* cm^{-1}; Anal calcd for $C_{34}H_{30}FeO_4S$ (590.51): C 68.92 H 5.44, S 5.43; found: C 68.85, H 5.44, S 5.47.

Dimethyl 2,5-di(2-naphthyl)-2-ferrocenyltetrahydrothiophene-3,3-dicarboxylate (9g): Yield: 25 mg (31%); mp = 220°C (dec.). ^{1}H-NMR (600 MHz, $CDCl_3$): δ 2.78 (dd, *J* = 13.8, *J* = 4.3 Hz, 1H, *H*C(4)), 3.44 (s, 3H, OCH_3), 3.51 (s, 3H, OCH_3), 3.56-3.58 (m, 1*H*C(Fc)), 3.76 (dd, *J* = 13.7, *J* = 12.9 Hz, 1H, *H*C(4)), 4.00-4.02 (m, 1*H*C(Fc)), 4.07 (s, 5*H*C(Fc)), 4.31-4.33 (m, 1*H*C(Fc)), 4.78-4.80 (m, 1*H*C(Fc)), 5.05 (dd, *J* = 12.6, *J* = 4.3 Hz, 1H, *H*C(5)), 7.52-7.58 (m, 4 arom. *H*C), 7.85-7.94 (m, 5 arom. *H*C), 7.96-8.01 (m, 2 arom. *H*C), 8.04 (s, 1 arom. *H*C), 8.38-8.41 (m, 1 arom. *H*C), 8.77-8.78 (m, 1 arom. *H*C); ^{13}C-NMR (151 MHz, $CDCl_3$): δ 48.1 (C(4)), 48.3 (C(5)), 52.5, 52,6 (2OCH_3), 67.8, 68.7, 69.2, 69.9, 71.2 (for 9H*C*(Fc)), 71.2, 73.5 (C(2) and C(3)), 97.3 (*C*(Fc)), 125.7, 125.8, 125.9, 126.1, 126.2, 126.3, 126.9, 127.3, 127.4, 127.7, 127.9, 128.0, 128.6, 128.7 (14 arom. H*C*), 132.1, 132.8, 133.1, 133.4, 136.1, 141.8 (6 arom. *C*), 168.9, 170.4 (2*C*=O); IR: ν 1723*vs* (2C=O), 1433*m*, 1299*m*, 1246*s*, 1217*m*, 1172*m*, 814*s*, 751*s*, 497*m*, 473*vs* cm^{-1}; Anal. calcd for $C_{38}H_{32}FeO_4S$ (640.57): C 71.25 H 5.04, S 5.01; found: C 71.21, H 5.25, S 5.01.

Dimethyl 2-ferrocenyl-2-phenyl-5-(4-(methyl)phenyl)tetrahydrothiophene-3,3-dicarboxylate (9h): Yield: 133 mg (85,1%). Yellow crystals; mp 146-148°C; ^{1}H-NMR (600 MHz, $CDCl_3$): δ 2.41 (s, 3H, CH_3), 2.62 (dd, *J* = 13.9, *J* = 4.3 Hz, 1H, *H*C(4)), 3.44 (s, 3H, OCH_3), 3.46 (s, 3H, OCH_3), 3.53-3.57 (dd, *J* = 13.6, *J* = 12.9 Hz, 1H, *H*C(4)), 3.57-3.58 (m, 1*H*C(Fc)), 4.00-4.02 (m, 1*H*C(Fc)), 4.06 (s, 5*H*C(Fc)), 4.27-4.29 (m, 1*H*C(Fc)), 4.68-4.70 (m, 1*H*C(Fc)), 4.78 (dd, *J* = 12.6, *J* = 4.3 Hz, 1H, *H*C(5)), 7.25, 7.52 (AB system, *J* = 8.0 Hz, 4 arom. *H*C), 7.33-7.34 (m, 1 arom. *H*C), 7.39-7.43 (m, 2 arom. *H*C), 8.21-8.24 (m, 2 arom. *H*C); ^{13}C-NMR (151 MHz, $CDCl_3$): δ = 21.2 (CH_3), 47.8 (C(5)), 48.4 (C(4)), 52.4 (2OCH_3), 67.8, 68.7, 69.2, 69.9, 71.0 (for 9H*C*(Fc)), 70.9, 73.7 (C(2) and C(3)), 97.0 (*C*(Fc)), 126.6, 127.1, 127.9, 128.8, 129.5 (for 9 arom. H*C*), 135.8, 137.6, 144.3 (3 arom. *C*), 168.9, 170.4 (2*C*=O); IR: ν 1729*brvs* (2C=O),

1429*m*, 1254*s*, 1162*s*, 1023*m*, 935*m*, 814*vs*, 721*s*, 700*s*, 484*m* cm^{-1}; Anal. calcd for $C_{31}H_{30}FeO_4S$ (554.48): C 67.15, H 5.45, S 5.78; found: C 67.13, H 5.61, S 5.73.

Dimethyl 2-ferrocenyl-2-phenyl-5-(4-(methoxyl)phenyl)tetrahydrothiophene-3,3-di-carboxylate (9i): Yield: 128 mg (79%). Yellow crystals; mp 138-140°C. ^{1}H-NMR (600 MHz, $CDCl_3$): δ 2.61 (dd, *J* = 13.9, *J* = 4.4 Hz, 1H, *H*C(4)), 3.44 (s, 3H, OCH_3), 3.45 (s, 3H, OCH_3), 3.54 (dd, *J* = 13.6, *J* = 12.9 Hz, 1H, *H*C(4)), 3.57-3.59 (m, 1*H*C(Fc)), 3.87 (s, 3H, OCH_3), 4.00-4.02 (m, 1*H*C(Fc)), 4.06 (s, 1*H*C(Fc)), 4.27-4.29 (m, 1*H*C(Fc)), 4.68-4.70 (m, 1*H*C(Fc)), 4.77 (dd, *J* = 12.6, *J* = 4.3 Hz, 1H, *H*C(5)), 6.98, 7.56 (AB system, *J* = 8.6 Hz, 4 arom. *H*C), 7.31-7.35 (m, 1 arom. *H*C), 7.39-7.44 (m, 2 arom. *H*C), 8.21-8.24 (m, 2 arom. *H*C); ^{13}C-NMR (151 MHz, $CDCl_3$): δ 47.5 (C(5)), 48.5 (C(4)), 52.4, 55.3 (for 3O*C*H_3), 67.8, 68.7, 69.2, 69.9, 71.0 (for 9H*C*(Fc)), 70.8, 73.6 (C(2) and C(3)), 96.9 (*C*(Fc)), 114.2, 126.5, 127.0, 128.8, 129.1, (for 9 arom. H*C*), 130.6, 144.3, 159.2 (3 arom. *C*), 168.9, 170.3 (2*C*=O); IR: ν 1731*brs* (2C=O), 1515*m*, 1431*m*, 1250*vs*, 1157*s*, 1039*m*, 935*m*, 833*m*, 818*m*, 721*m*, 700*m*, 486*s* cm^{-1}; Anal. calcd for $C_{31}H_{30}FeO_5S$ (570.48): C 65.27, H 5.30, S 5.62; found: C 65.35, H 5.42, S 5.61.

Dimethyl 2-ferrocenyl-2-phenyl-5-(4-(bromo)phenyl)tetrahydrothiophene-3,3-dicarboxy-late (9j): Yield: 128 mg (92,5%). Yellow crystals; mp 182-184°C. ^{1}H-NMR (600 MHz, $CDCl_3$): δ 2.63 (dd, *J* = 13.9, *J* = 4.4 Hz, 1H, *H*C(4)), 3.44 (s, 3H, OCH_3), 3.45 (s, 3H, OCH_3), 3.49 (dd, *J* = 13.9, *J* = 12.7 Hz, 1H, *H*C(4)), 3.57-3.59 (m, 1*H*C(Fc)), 4.00-4.02 (m, 1*H*C(Fc)), 4.06 (s, 5*H*C(Fc)), 4.27-4.29 (m, 1*H*C(Fc)), 4.61-4.63 (m, 1*H*C(Fc)), 4.76 (dd, *J* = 12.6, *J* = 4.3 Hz, 1H, *H*C(5)), 7.31-7.35 (m, 1 arom. *H*C), 7.39-7.43 (m, 2 arom. *H*C), 7.50, 7.56 (AB system, *J* = 8.4 Hz, 4 arom. *H*C), 8.20-8.22 (m, 2 arom. *H*C); ^{13}C-NMR (151 MHz, $CDCl_3$): δ 47.4 (C(5)), 48.2 (C(4)), 52.4, 52.5 (2O*C*H_3), 67.8, 68.8, 69.2, 69.7, 71.1 (for 9H*C*(Fc)), 71.2, 73.5 (C(2) and C(3)), 96.7 (*C*(Fc)), 126.6, 127.1, 128.7, 129.7, 131.9 (for 9 arom. H*C*), 121.6, 138.0, 144.0 (3 arom. *C*), 168.7, 170.2 (2*C*=O); IR: ν 1729*brs* (2C=O), 1490*m*, 1440*m*, 1256*s*, 1162*s*, 1073*m*, 814*s*, 721*m*, 700*m*, 486*m* cm^{-1}; Anal. calcd for $C_{30}H_{27}BrFeO_4S$ (619.35): C 58.18, H 4.39, S 5.18; found: C 58.11, H 4.41, S 5.13.

Dimethyl 2-ferrocenyl-2-phenyl-5-(4-(trifluoromethyl)phenyl)tetrahydro-thiophene-3,3-dicarboxylate (9k): Yield: 134 mg (95,1%). Yellow crystals; mp 168-170°C. ^{1}H-NMR (600 MHz, $CDCl_3$): δ 2.68 (dd, *J* = 13.9, *J* = 4.4 Hz, 1H, *H*C(4)), 3.45 (s, 3H, OCH_3), 3.47 (s, 3H, OCH_3), 3.53 (dd, *J* = 13.8, *J* = 12.7 Hz, 1H, *H*C(4)), 3.59-3.61 (m, 1*H*C(Fc)), 4.02-4.04 (m, 1*H*C(Fc)),

4.07 (s, 5*H*C(Fc)), 4.29-4.31 (m, 1*H*C(Fc)), 4.61-4.63 (m, 1*H*C(Fc)), 4.86 (dd, *J* = 12.6, *J* = 4.4 Hz, 1H, *H*C(5)), 7.33-7.37 (m, 1 arom. *H*C), 7.41-7.45 (m, 2 arom. *H*C), 7.70, 7.75 (AB system, *J* = 8.2 Hz, 4 arom. *H*C), 8.20-8.23 (m, 2 arom. *H*C); ^{13}C-NMR (151 MHz, $CDCl_3$): δ 47.5 (C(5)), 48.2 (C(4)), 52.5, 52.6 (2O*C*H_3), 67.8, 68.8, 69.2, 69.7, 71.7 (for 9H*C*(Fc)), 71.3, 73.6 (C(2) and C(3)), 96.6 (*C*(Fc)), 125.7, 125.8, 126.7, 127.1, 128.4, 128.7 (for 9 arom. H*C*), 129.7, 129.9, 130.2, 130.4 (*C*F_3), 143.2, 143.9 (2 arom. *C*), 168.6, 170.1 (2*C*=O); IR: ν 1731*brs* (2C=O), 1429*m*, 1328*m*, 1258*m*, 1162*s*, 1116*s*, 1067*s*, 936*m*, 820*m*, 723*m*, 700*m*, 486*m* cm^{-1}; Anal. calcd for $C_{31}H_{27}F_3FeO_4S$ (608.45): C 61.19, H 4.47, S 5.27; found: C 61.26, H 4.48, S 5.17.

Dimethyl 2-ferrocenyl-2-(2-furanyl)-5-phenyltetrahydrothiophene-3,3-dicarboxylate (9m): Yield: 152 mg (96%). Isolated chromatographically as a 60:40 mixture of isomers. Yellow crystals; mp 124-126°C. Major isomer (based on the registered spectrum of the mixture): ^{1}H-NMR: δ 3.04 (dd, *J* = 13.8, *J* = 6.4 Hz, 1H, *H*C(4)), 3.40 (dd, *J* = 11.2, *J* = 3.2 Hz, 1H, *H*C(4)), 3.49 (s, 3H, OCH_3), 3.59 (s, 3H, OCH_3), 5.24 (dd, *J* = 11.2, *J* = 6.4 Hz, 1H, *H*C(5)); Minor isomer (based on the registered spectrum of the mixture): ^{1}H-NMR: δ 2.74 (dd, *J* = 13.8, 5.2 Hz, 1H, *H*C(4)), 3.42 (dd, *J* = 12.1, *J* = 4.0 Hz, 1H, *H*C(4)), 3.48 (s, 3H, OCH_3), 3.64 (s, 3H, OCH_3), 4.79 (dd, *J* = 12.0, *J* = 5.2 Hz, 1H, *H*C(5)); Remaining signals registered for the mixture: ^{1}H-NMR *δ* 4.09 (s, 4*H*C(Fc)), 4.10-4.11 (s, 1*H*C(Fc)), 4.12 (s, 6*H*C(Fc)), 4.23-4.26 (m, 4*H*C(Fc)), 4.55-4.57 (m, 1*H*C(Fc)), 4.69-4.71 (m, 1*H*C(Fc)), 4.55-4.57 (m, 1*H*C(Fc)), 6.48-6.50 (m, 2 arom. *H*C), 6.84 (d, *J* = 3 Hz, 1 arom. *H*C), 7.16 (d, *J* = 3.1 Hz, 1 arom. *H*C), 7.34-7.38 (m, 3 arom. *H*C), 7.40-7.45 (m, 2 arom. *H*C), 7.47-7.49 (m, 1 arom. *H*C), 7.49-7.51 (m, 1 arom. *H*C), 7.52-7.55 (m, 3 arom. *H*C), 7.59-7.62 (m, 2 arom. *H*C); ^{13}C-NMR (registered for the mixture): *δ* 46.2, 47.7 (2C(4)); 48.1, 49.5 (2HC(5)); 52.3, 52.4, 52.6, 52.8 (4O*C*H_3); 64.0, 64.2 (2C(2), 71.8, 72.0 (2C(3)); 66.8, 68.1, 68.4, 68.9, 69.3, 69.5, 69.9, 70.1, 70.4 71.9 (for 18H*C*(Fc)); 86.0, 92.5 (2*C*(Fc)); 108.1, 109.2, 110.5, 110.6, 140.5, 140.8 (6 arom. H*C*); 127.4, 127.8, 128.0, 128.1, 128.6, 128.7 (for 10 arom. H*C*), 138.9, 141.3, 156.3, 157.9 (4 arom. *C*), 168.4, 168.8, 169.0, 169.4 (4*C*=O); IR (registered for the mixture): ν 1731*vs* (2C=O), 1492*m*, 1429*m*, 1248*s*, 1146*m*, 816*m*, 732*m*, 698*vs*, 598*m*, 490*vs* cm^{-1}; HRMS–EI (*m*/*z*): $[M]^+$ calcd for $[C_{28}H_{26}FeO_5S]^+$, 530.0850; found 530.0856.

Dimethyl 2-ferrocenyl-5-(phtalimid-1-yl)-2-phenyltetrahydrothiophene-3,3-dicarboxylate (9n): Yield: 48 mg (34,1%). Isolated chromatographically as a 60:40 mixture of isomers. Yellow crystals; mp ca. 170°C (dec.); Major isomer (based on the registered spectrum of the mixture):

^{1}H-NMR: δ 2.47 (dd, *J* = 13.7, *J* = 5.1 Hz, 1H, *H*C(4)), 3.44 (s, 3H, OCH_3), 3.46 (s, 3H, OCH_3), 4.66 (pseudo-dd, *J* = 13.5, *J* = 12.3 Hz, 1H, *H*C(4)), 6.31 (dd, *J* = 12.1, *J* = 5.1 Hz, 1H, *H*C(5)); Minor isomer (based on the registered spectrum of the mixture): ^{1}H-NMR: δ 3.22 (dd, *J* = 14.5, *J* = 7.7 Hz, 1H, HC(4)), 3.49 (s, 3H, OCH_3), 3.56 (s, 3H, OCH_3), 4.23 (pseudo-dd, *J* = 14.5 Hz, *J* = 7.5, Hz, 1H, *H*C(4)), 6.43 (pseudo-dd, *J* = 8.0, *J* = 7.4 Hz, 1H, *H*C(5)); Remaining signals registered for the mixture: δ 3.54 (s, 1*H*C(Fc)), 3.90 (s, 1*H*C(Fc)), 3.99 (s, 1*H*C(Fc)), 4.05 (s, 5*H*C(Fc)), 4.08 (s, 6*H*C(Fc)), 4.18 (s, 1*H*C(Fc)), 4.25-4.26 (m, 1*H*C(Fc)), 4.37 (s, 1*H*C(Fc)), 5.45 (s, 1*H*C(Fc)), 7.30-7.35 (m, 2 arom. *H*C), 7.37-7.44 (m, 3 arom. *H*C), 7.71-7.75 (m, 2 arom. *H*C), 7.79-7.83 (m, 2 arom. *H*C), 7.84-7.86 (m, 2 arom. *H*C), 7.94-7.98 (m, 2 arom. *H*C), 8.18-8.24 (m, 2 arom. *H*C); ^{13}C-NMR (registered for the mixture): δ 39.2, 40.2 (2C(4)), 52.3, 52.5, 52.6, 52.7 (4O*C*H_3), 53.1, 55.7 (2*H*C(5)), 70.9, 71.7, 72.5 (for 2C(2) and 2C(3)), 67.6, 69.1, 69.4, 69.5, 70.1, 71.4 (for 18*H*C(Fc)), 97.1 (for 2*C*(Fc)), 123.4, 123.6, 126.7, 126.8, 127.0, 127.1, 128.8, 129.1, 134.2, 134.4 (for 18 arom. H*C*), 131.8, 131.9, 143.9 (for 6 arom. *C*), 167.5, 167.7, 168.1, 168.9, 169.2, 170.1 (for 8*C*=O); **IR:** 1736*m*, 1716*vs* (2C=O), 1431*m*, 1354*m*, 1241*s*, 1172*m*, 1107*m*, 967*m*, 818*m*, 713*vs*, 501*s*, 486*s* cm^{-1}; Anal. calcd for $C_{32}H_{27}FeNO_6S$ (609.47): C 63.06, H 4.47, N 2.30, S 5.26; found: C 63.06, H 4.45, N 2.56, S 5.19.

2. Crystal structure determinations

Crystals were mounted in inert oil on nylon loops and transferred to the cold gas stream of the diffractometer (*trans*-**9c**: Oxford Diffraction Nova A using mirror-focussed Cu Kα radiation; *cis*-**9d**: Rigaku/Oxford XtaLAB Synergy using mirror-focussed MoKα radiation). Absorption corrections were implemented on the basis of multi-scans. The structures were refined anisotropically on F^2 using the program SHELXL-2017 [S1]. Hydrogen atoms were included using rigid methyl groups or a riding model starting from calculated positions.

Crystallographic data are summarized in Table S1. Additionally, complete data have been deposited with the Cambridge Crystallographic Data Centre under the numbers CCDC 1992864 & 1992865. Copies of the data can be obtained free of charge from www.ccdc.cam.ac.uk/data_request/cif.

Table S1: Crystallographic data and structure refinement details for compounds *trans*-**9c** and *cis*-**9d**.

Compound	*trans*-**9c**	*cis*-**9d**
CCDC number	1992864	1992865
Formula	$C_{25}H_{26}FeO_4S$	$C_{34}H_{30}FeO_4S$
M_r	478.37	590.59
Crystal size (mm)	0.2 x 0.03 x 0.01	0.15 x 0.1 x 0.05
Crystal system	monoclinic	triclinic
Space group	$P2_1/c$	P(-1)
Temperature (°C)	-173	-170
a (Å)	13.1715(5)	9.6177(2)
b (Å)	17.8170(5)	11.0241(3)
c (Å)	9.6616(4)	14.8441(3)
α (°)	90	71.105(2)
β (°)	106.781(4)	77.475(2)
γ (°)	90	65.076(3)
V (Å^3)	2170.80	1344.43
Z	4	2
D_x (Mg m^{-3})	1.464	1.459
λ (Å)	1.54184	0.71073
μ (mm^{-1})	6.7	0.68
Transmissions	0.638 – 1.000	0.929 – 1.000
F(000)	1000	616
$2\theta_{max}$	154.8	70.3
Refl. measured	46122	97549
Refl. indep.	4568	11091
R_{int}	0.055	0.037
Parameters	283	363
$wR(F^2$, all refl.)	0.095	0.080
$R(F, >4\sigma(F))$	0.037	0.029
S	1.04	1.06
Max. Δp (e Å^{-3})	0.38, –0.51	0.57, –0.40

3. ^{1}H and ^{13}C NMR spectra

3. 1. ^{1}H and ^{13}C NMR spectra for ferrocenyl (naphth-2-yl) thioketone (8c)

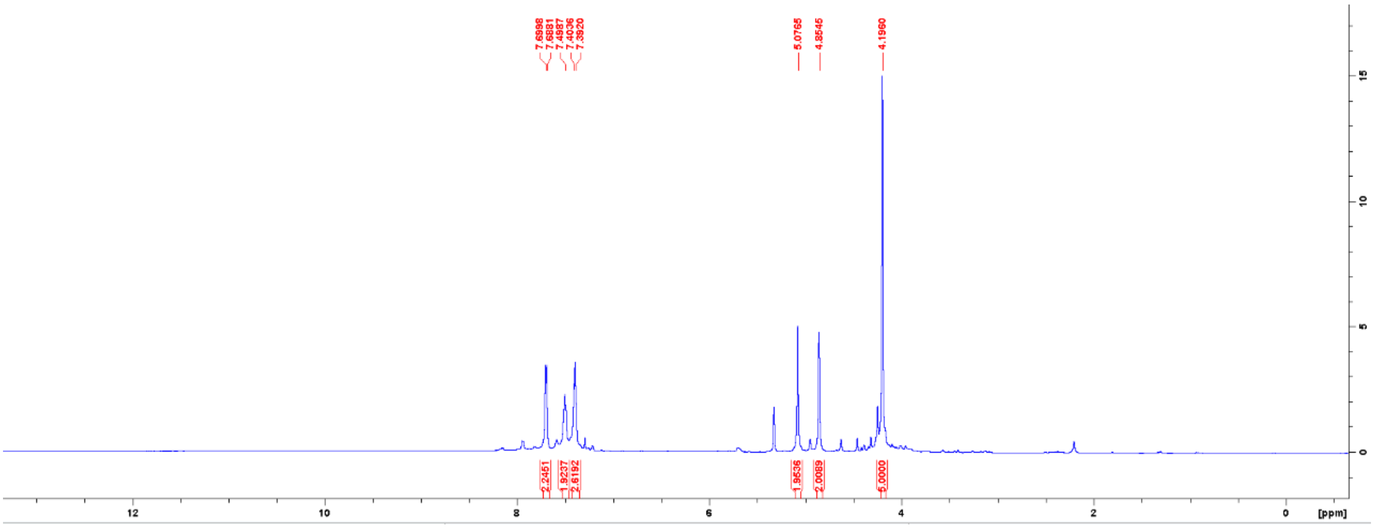

Figure S1: ^{1}H NMR spectrum for thioketone **8c**.

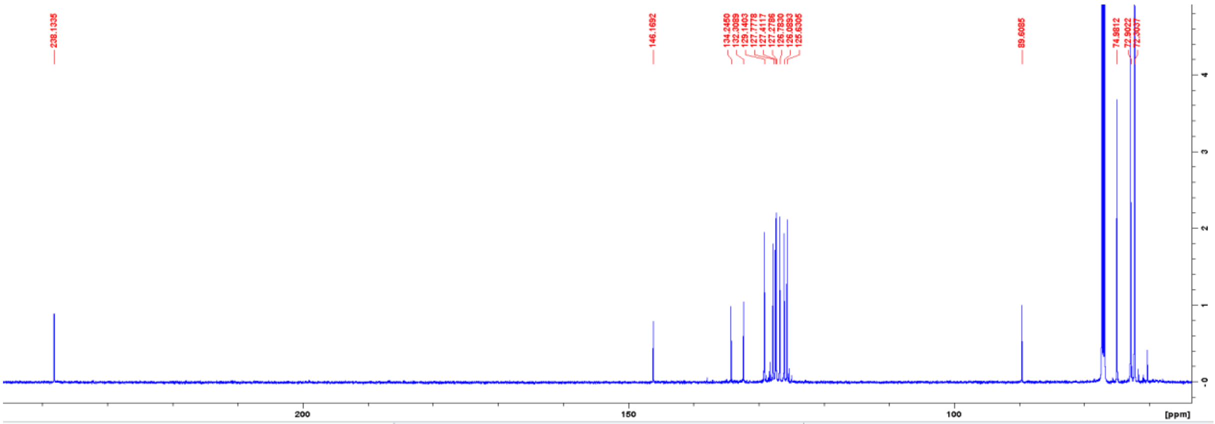

Figure S2: ^{13}C NMR spectrum for thioketone **8c**.

3.2. Collection of spectra for isolated tetrahydrothiophenes 9a–n

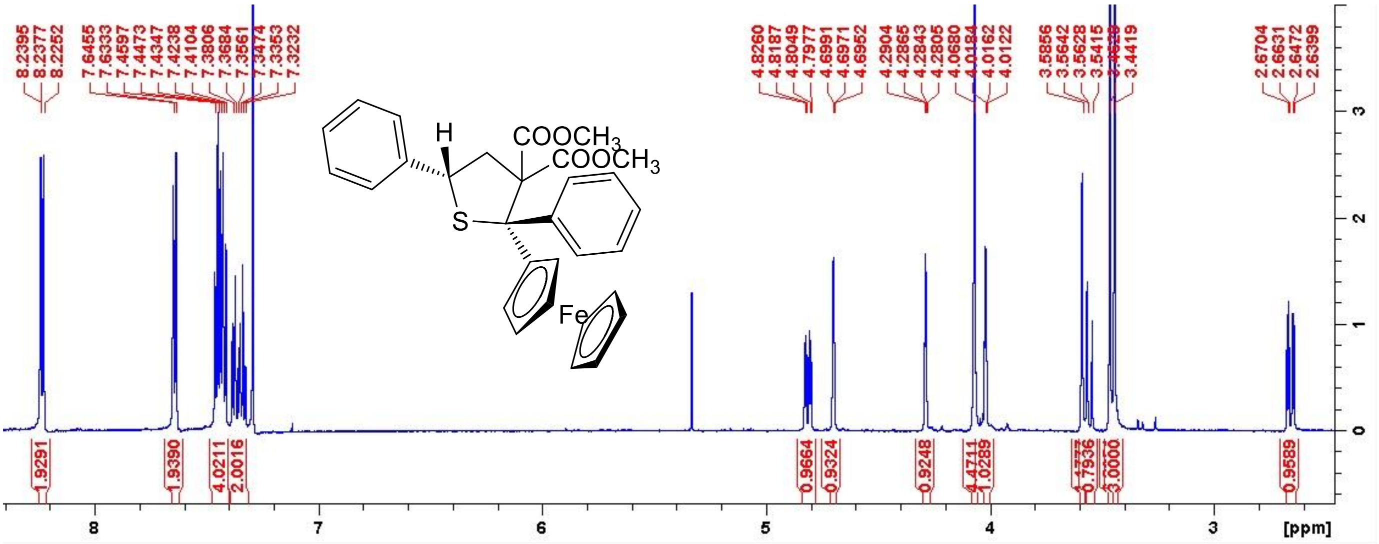

Figure S3: ^{1}H NMR spectrum for **9a**.

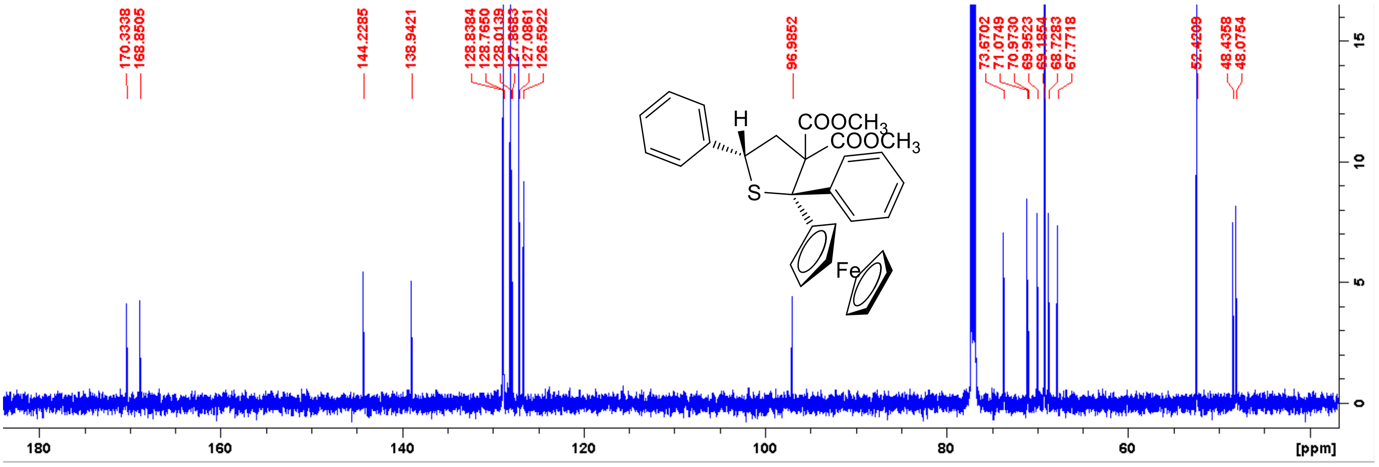

Figure S4: ^{13}C NMR spectrum for **9a**.

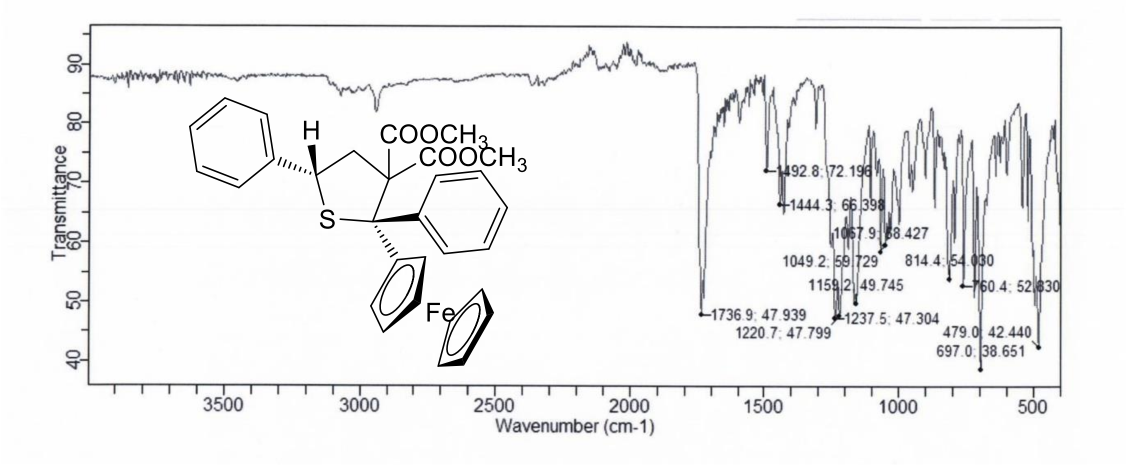

Figure S5: IR spectrum for **9a**.

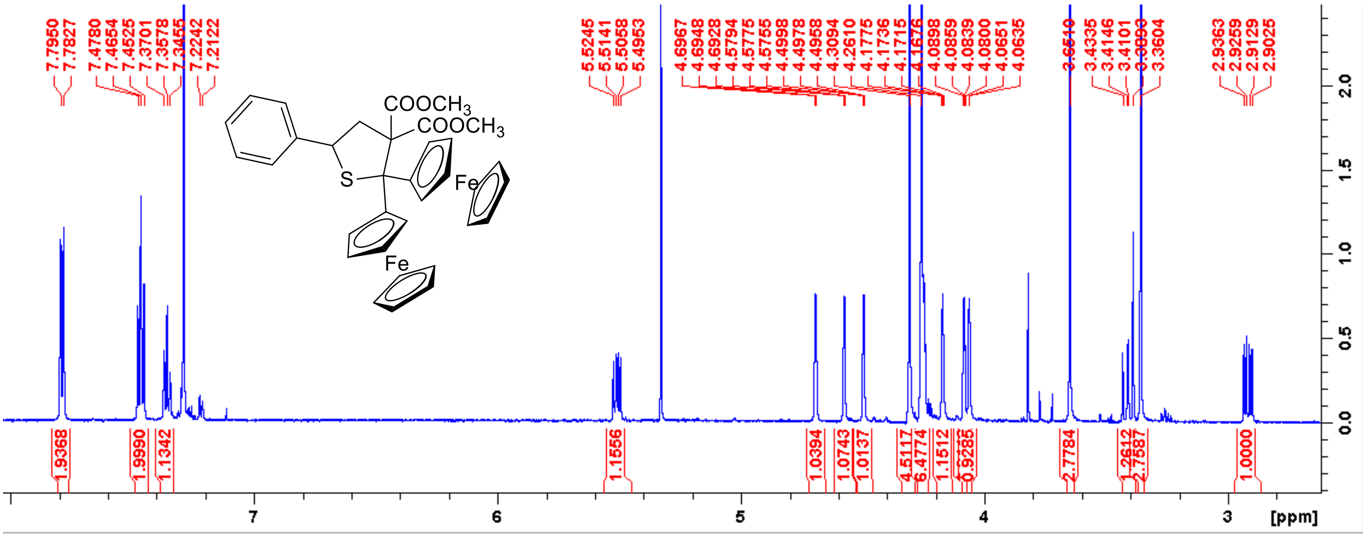

Figure S6: ^{1}H NMR spectrum for **9b**.

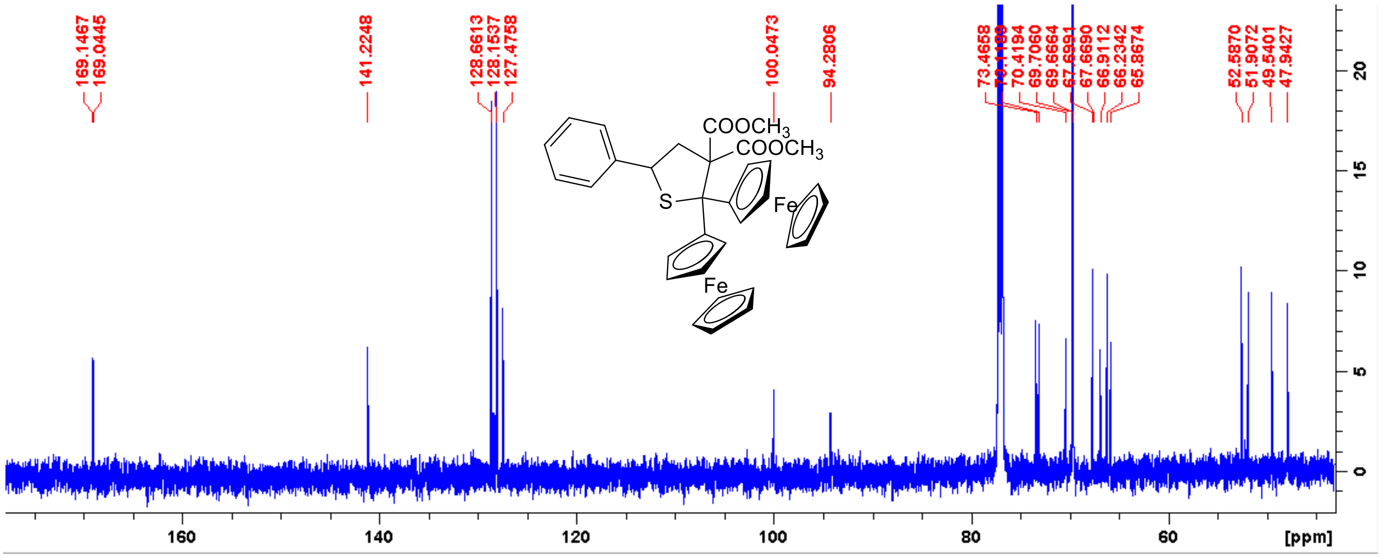

Figure S7: ^{13}C NMR spectrum for **9b**.

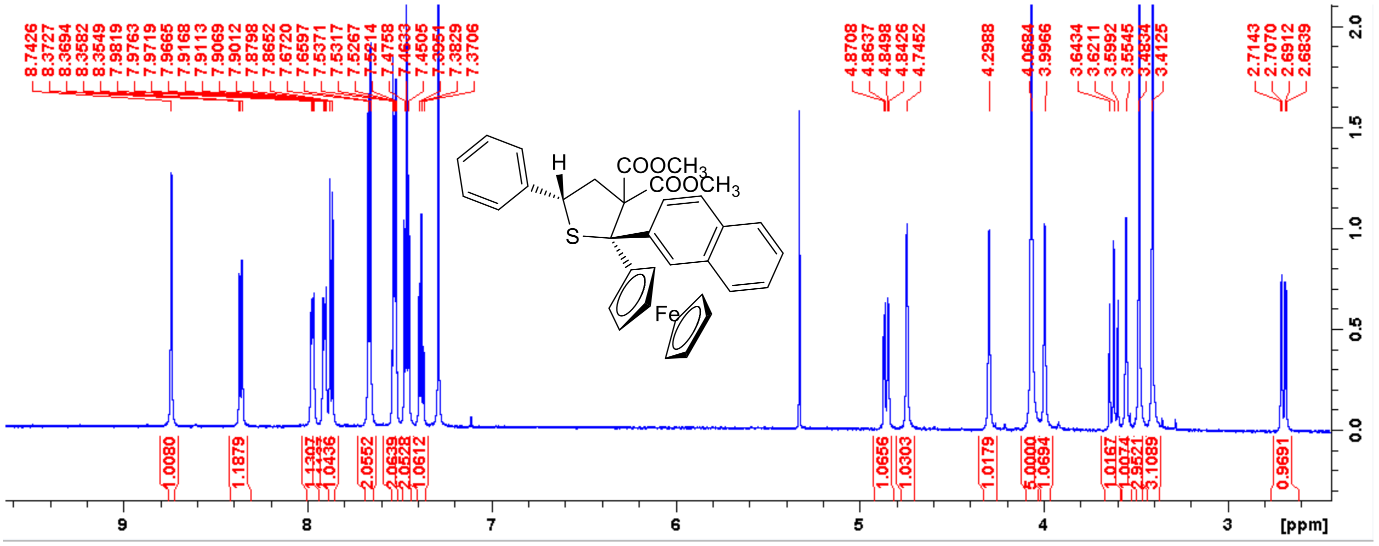

Figure S8: ^{1}H NMR spectrum for **9c**.

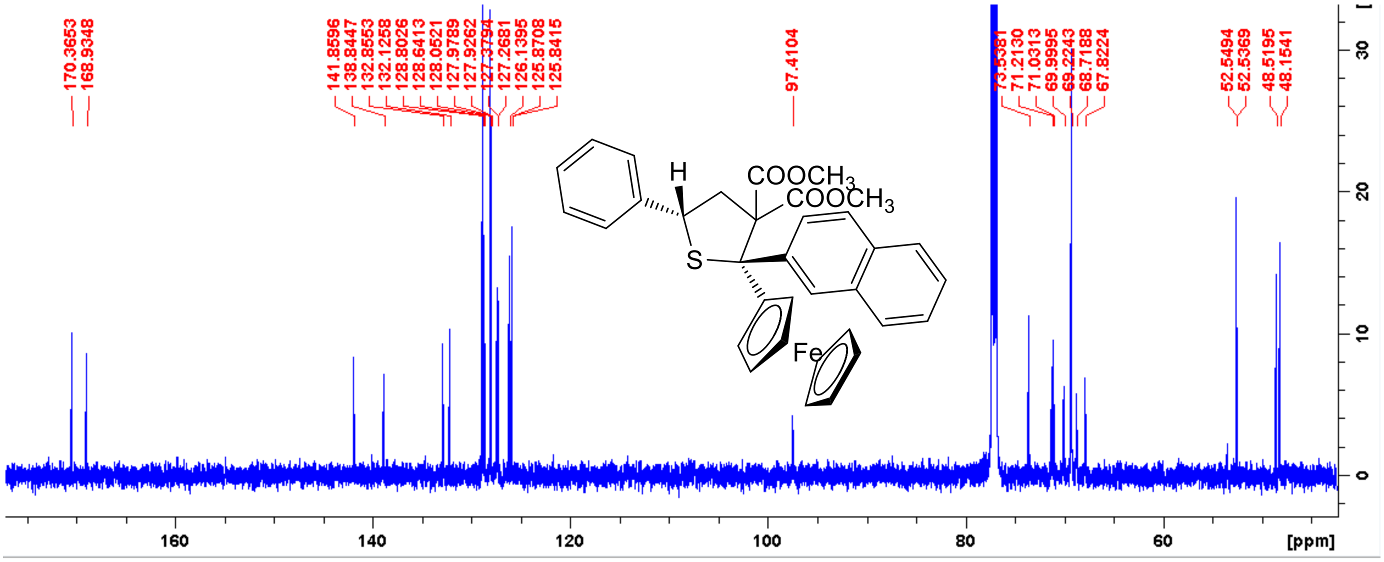

Figure S9: ^{13}C NMR spectrum for **9c**.

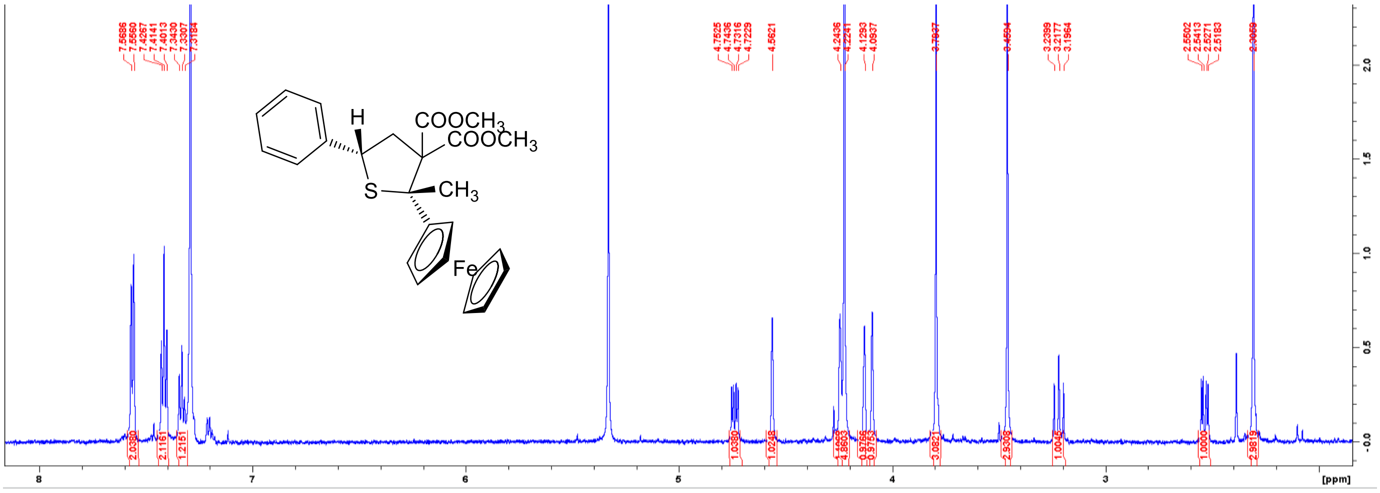

Figure S10: ^{1}H NMR spectrum for **9d** (isomer *cis*).

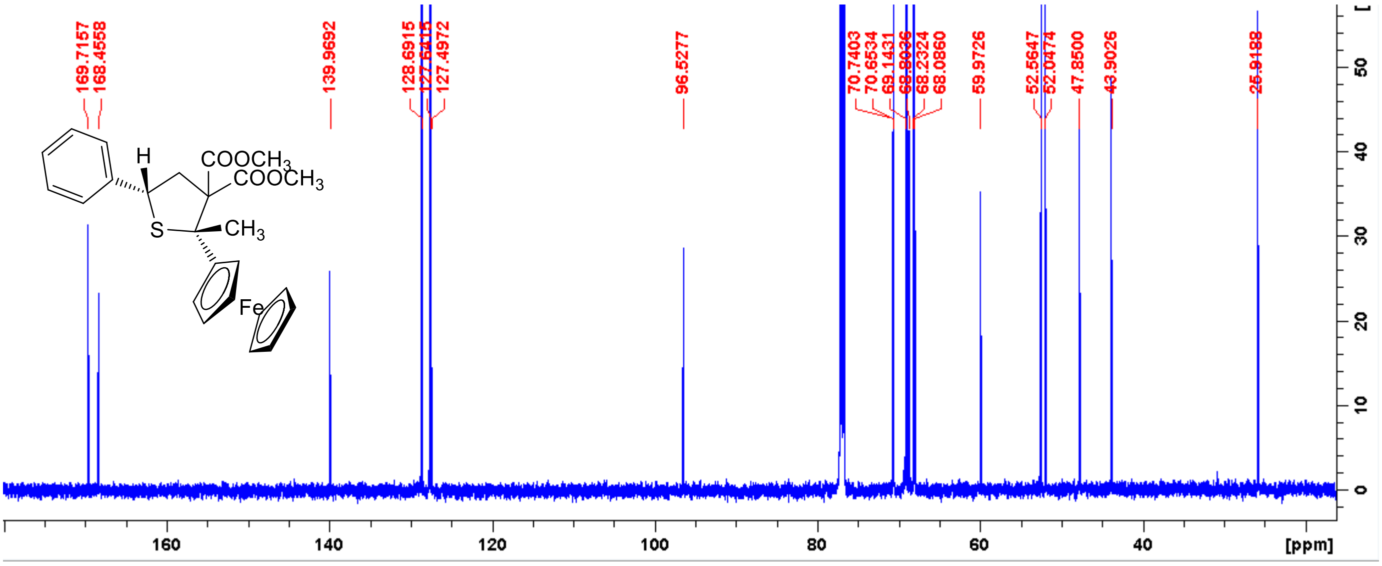

Figure S11: ^{13}C NMR spectrum for **9d** (isomer *cis*).

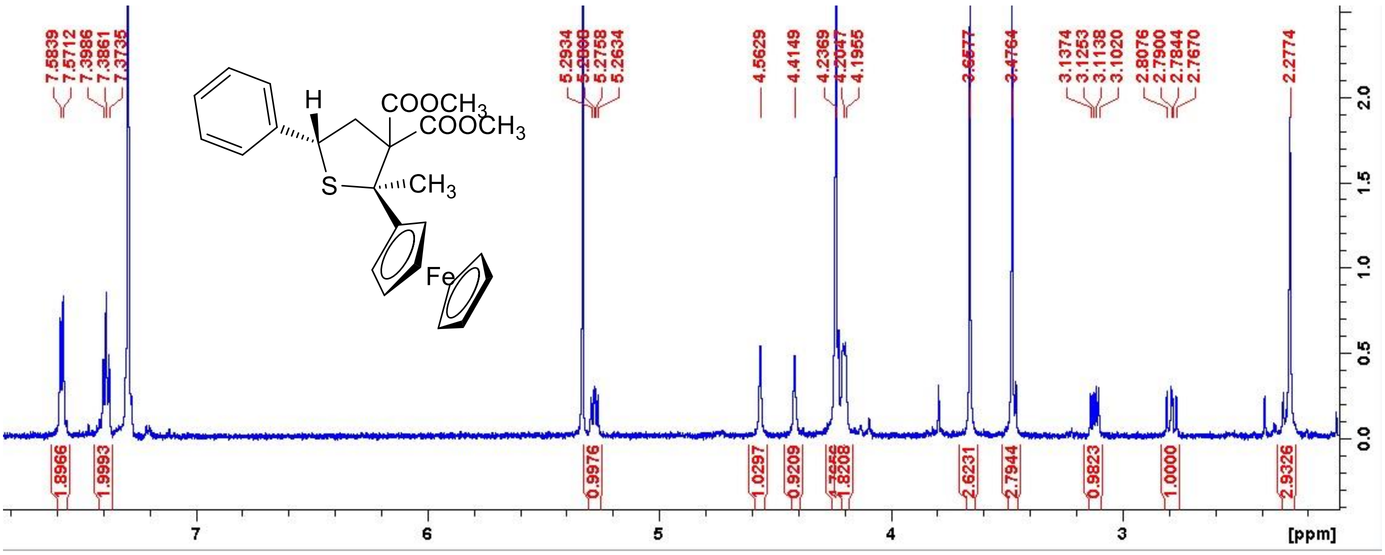

Figure S12: ^{1}H NMR spectrum for **9d** (isomer *trans*).

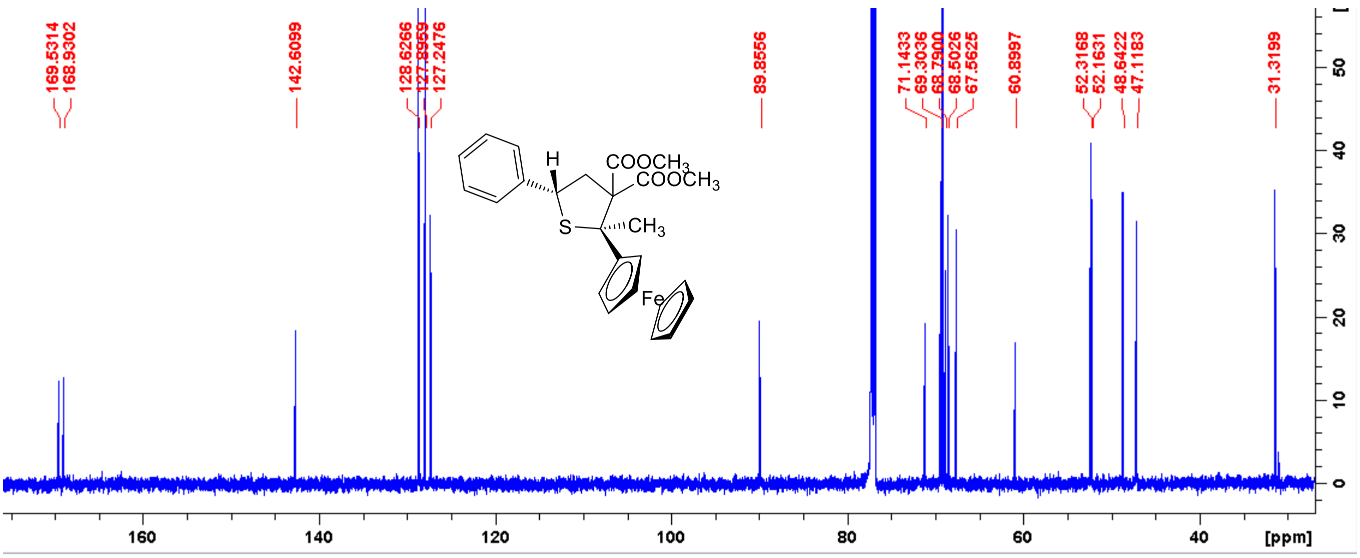

Figure S13: ^{13}C NMR spectrum for **9d** (isomer *trans*).

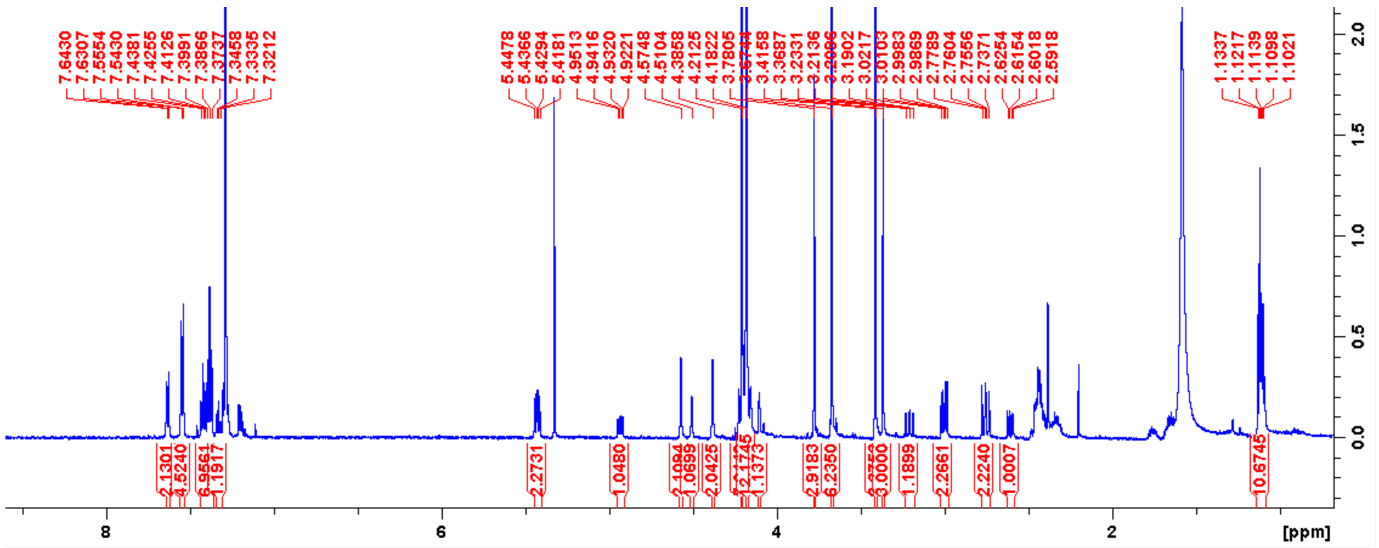

Figure S14: ^{1}H NMR spectrum for **9e** (crude mixture).

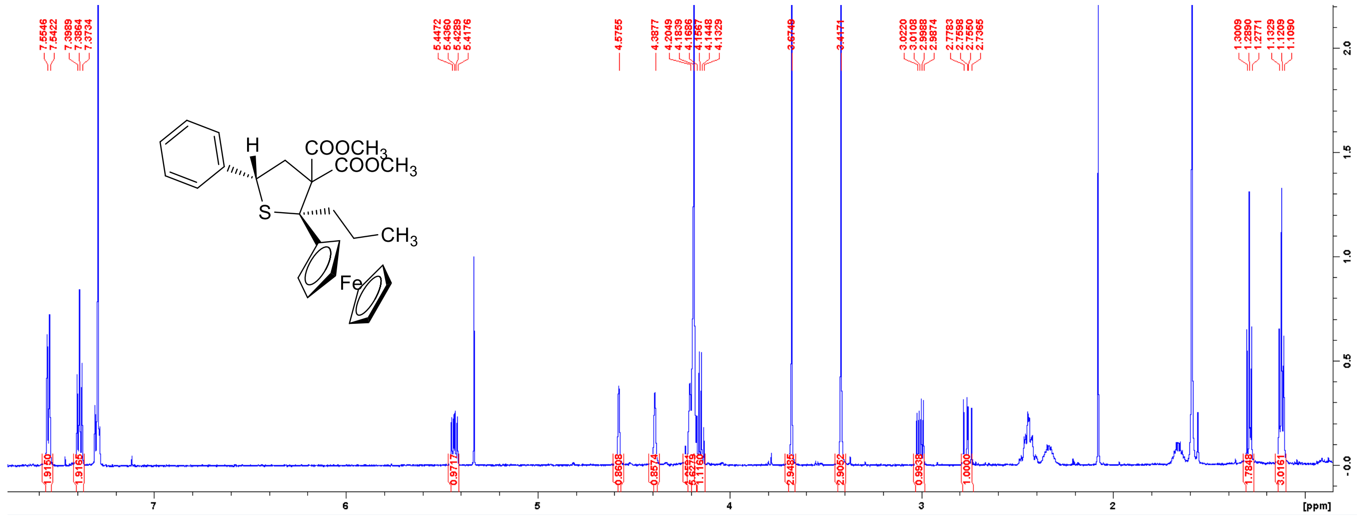

Figure S15: ^{1}H NMR spectrum for **9e** (isomer *trans*).

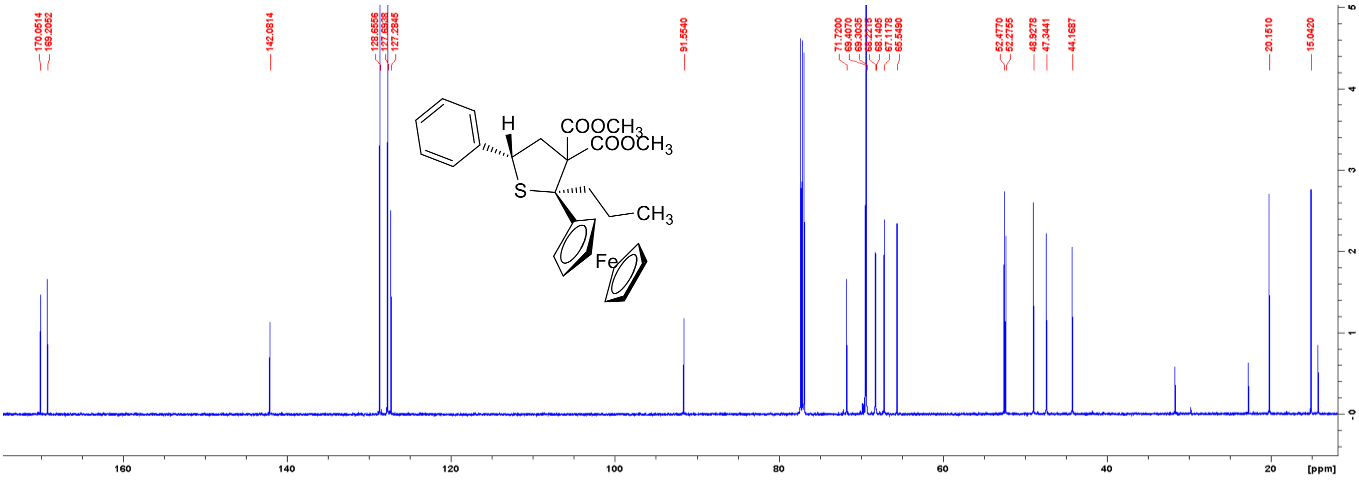

Figure S16: ^{13}C NMR spectrum for **9e** (isomer *trans*).

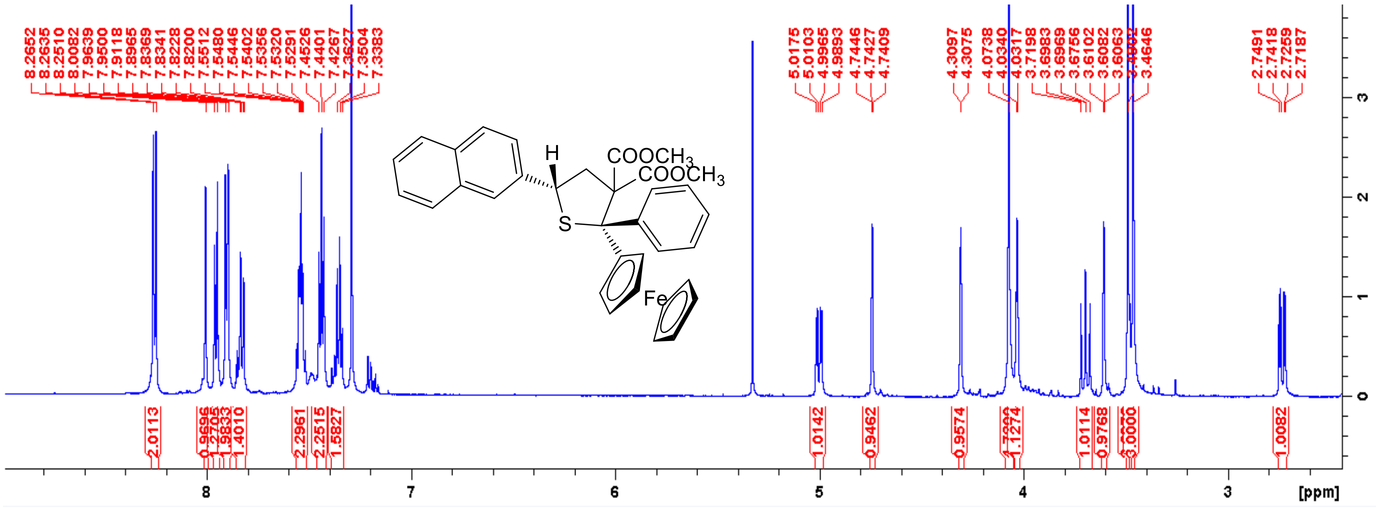

Figure S17: ^{1}H NMR spectrum for **9f**.

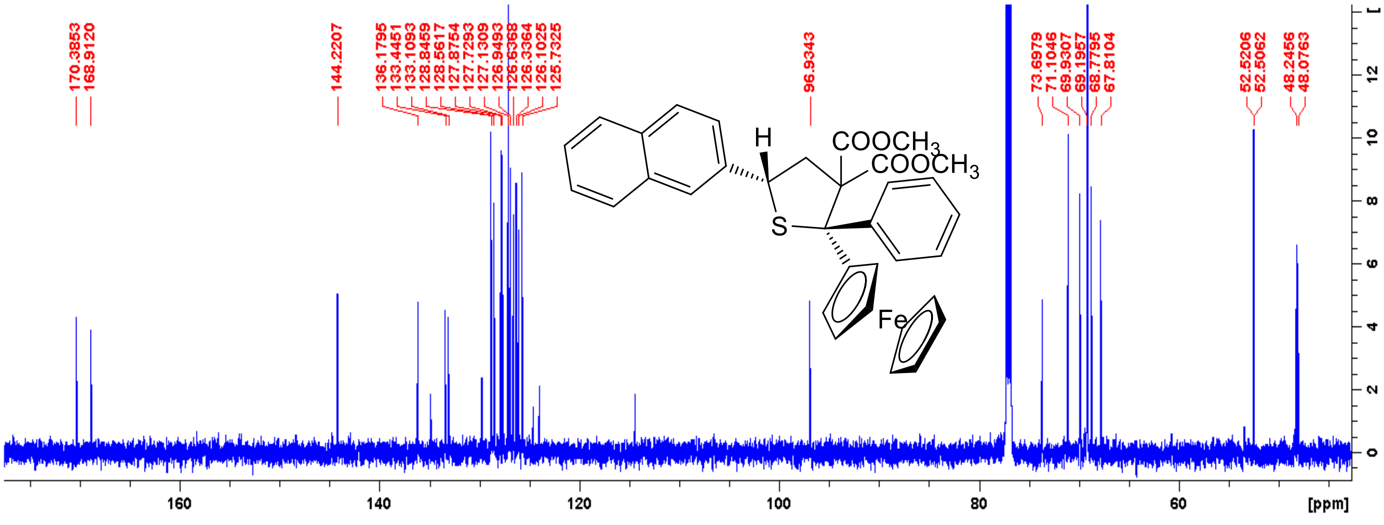

Figure S18: ^{13}C NMR spectrum for **9f**.

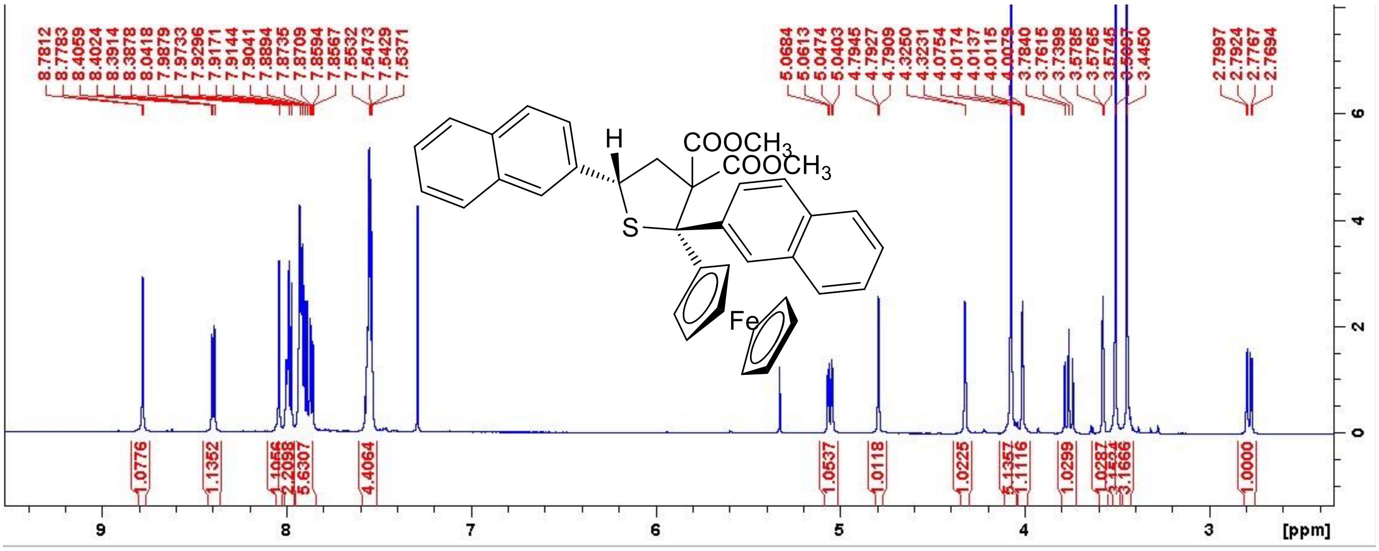

Figure S19: ^{1}H NMR spectrum for **9g**.

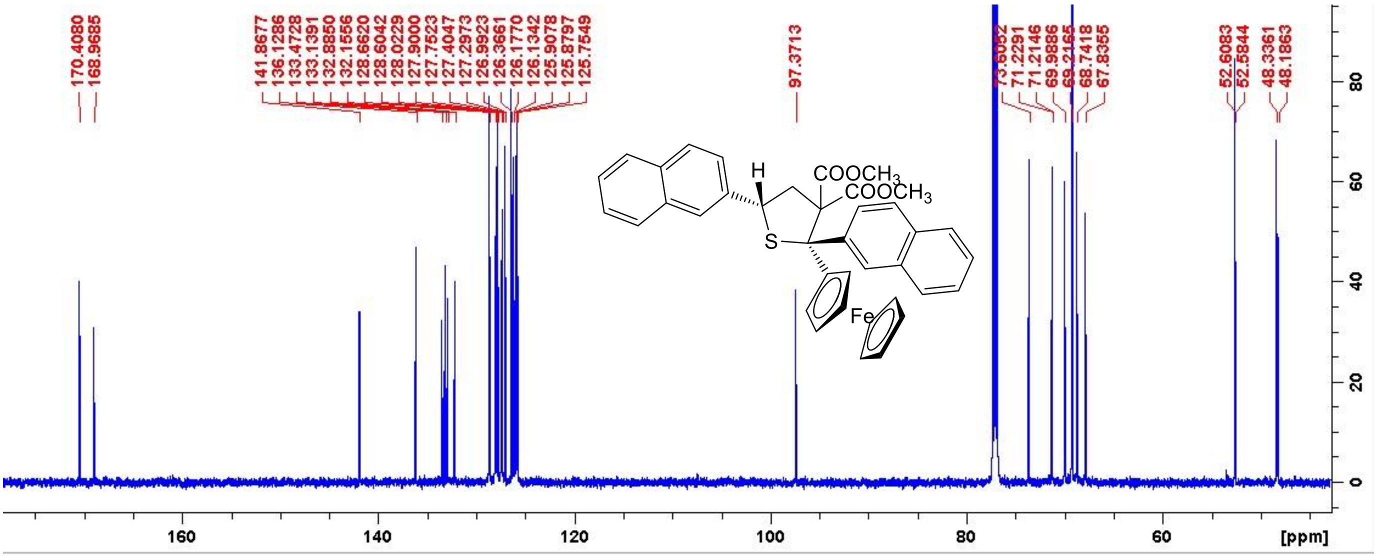

Figure S20: ^{13}C NMR spectrum for **9g**.

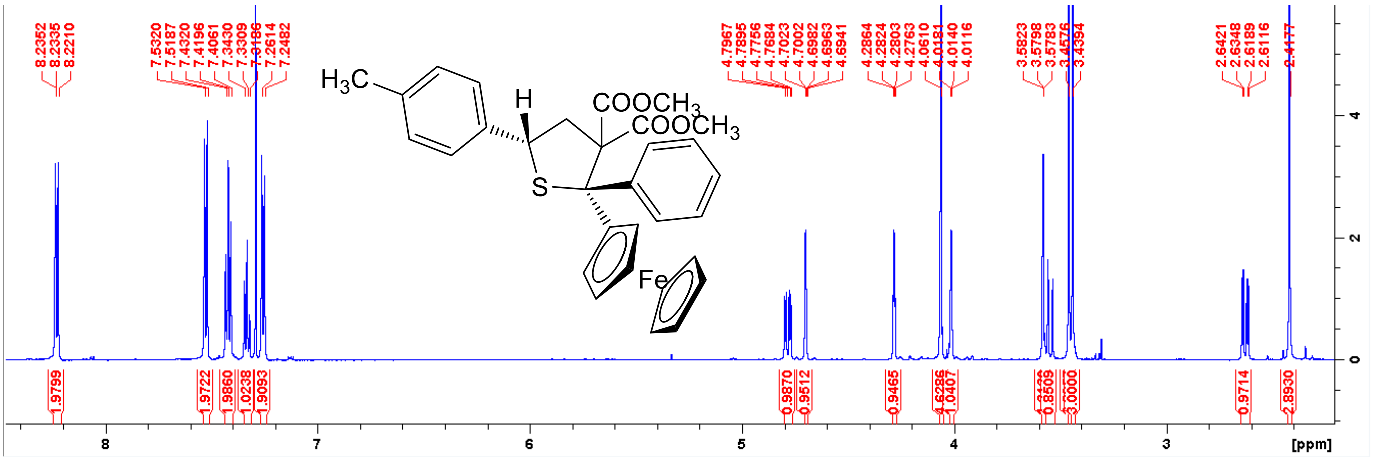

Figure S21: ^{1}H NMR spectrum for **9h**.

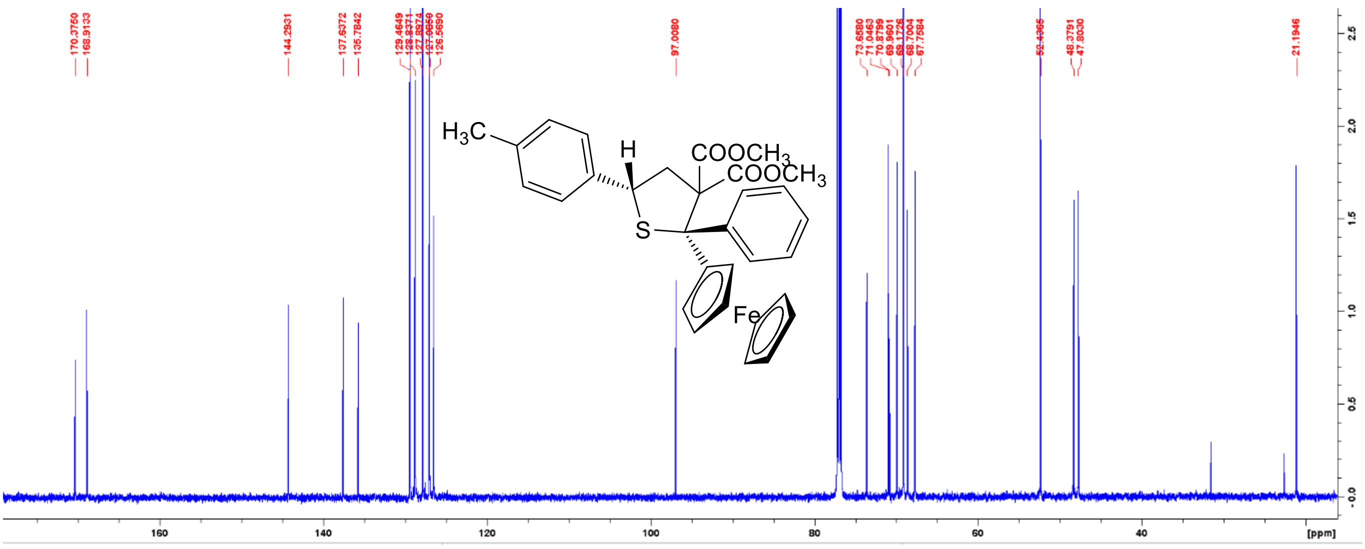

Figure S22: ^{13}C NMR spectrum for **9h**.

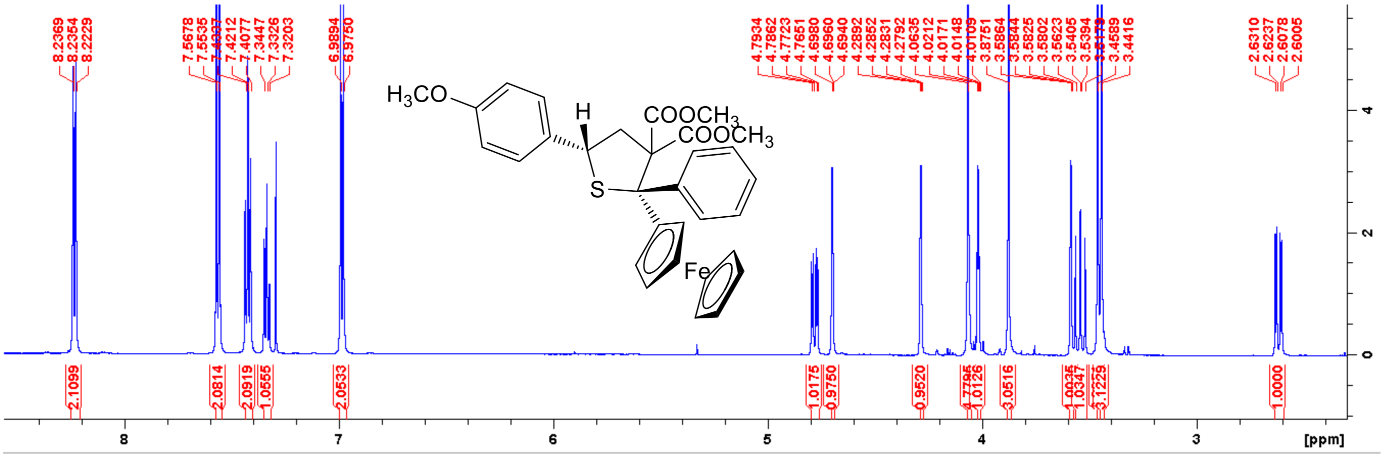

Figure S23: ^{1}H NMR spectrum for **9i**.

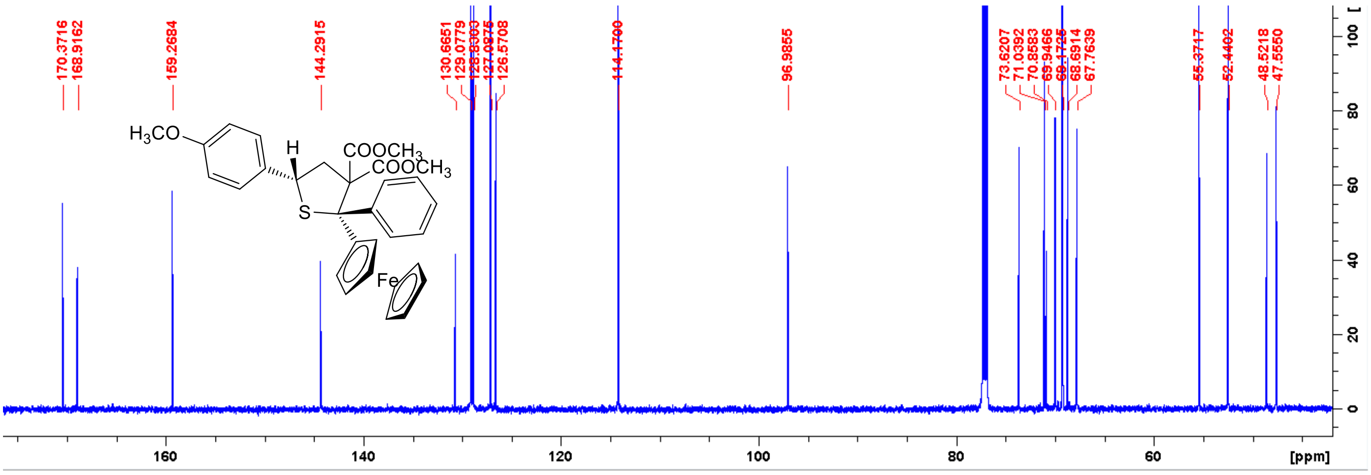

Figure S24: ^{13}C NMR spectrum for **9i**.

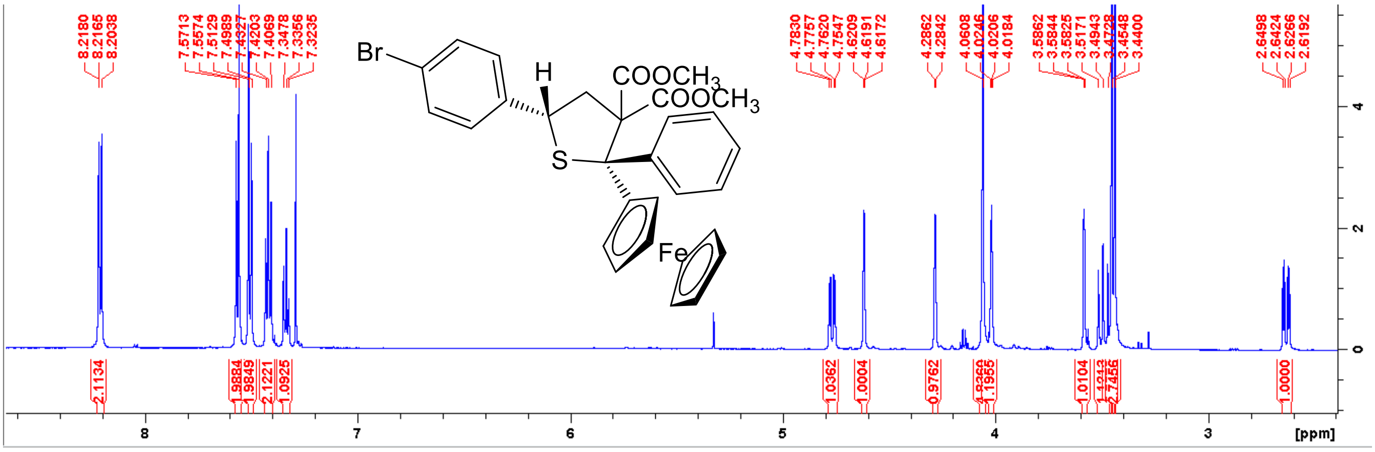

Figure S25: ^{1}H NMR spectrum for **9j**.

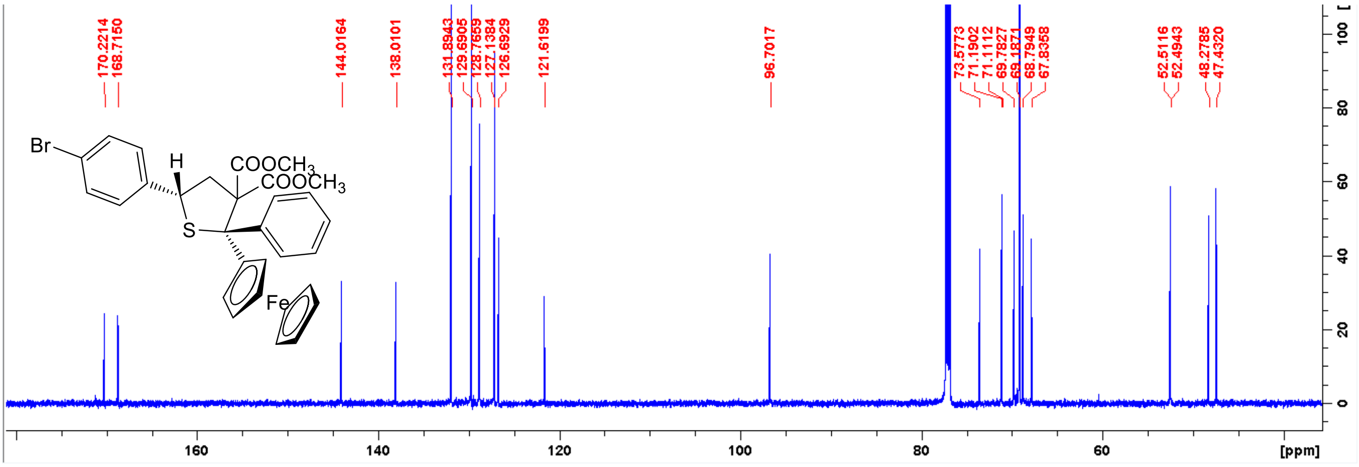

Figure S26: ^{13}C NMR spectrum for **9j**.

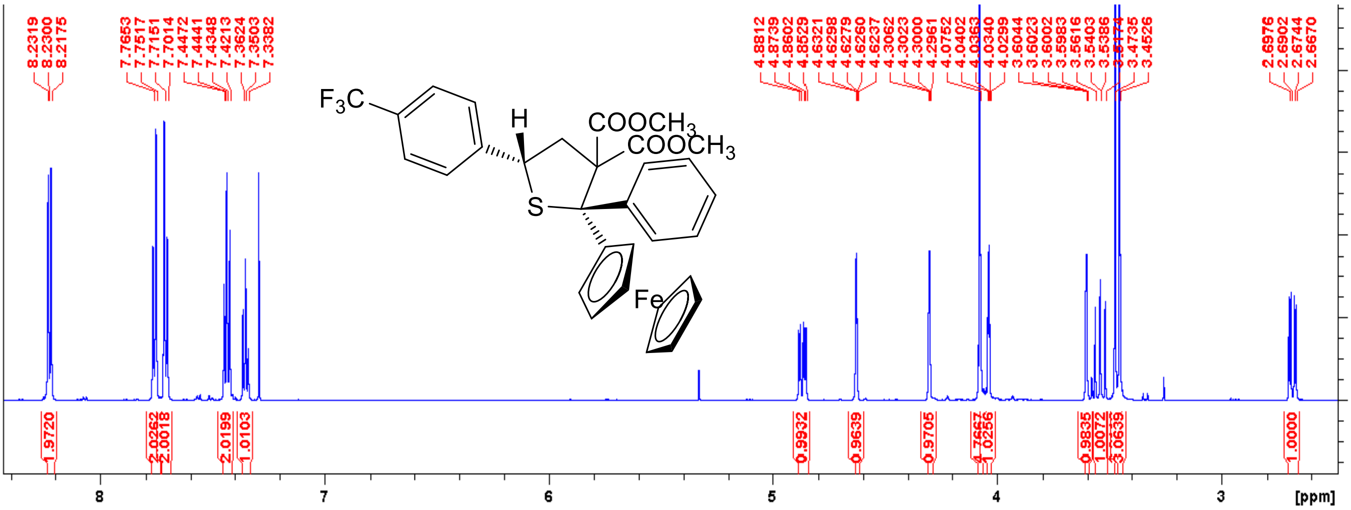

Figure S27: ^{1}H NMR spectrum for **9k**.

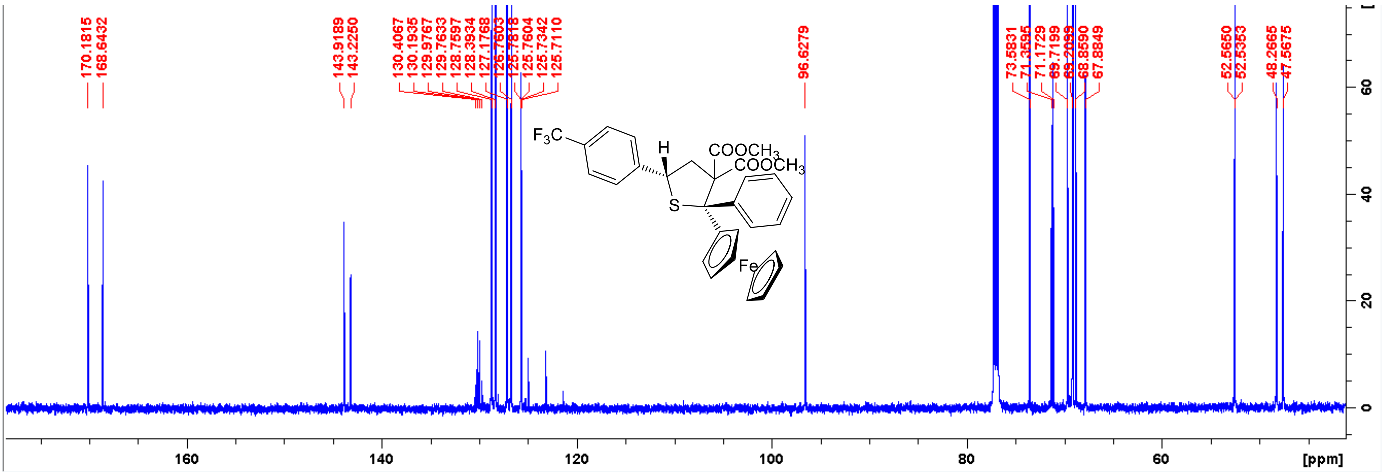

Figure S28: ^{13}C NMR spectrum for **9k**.

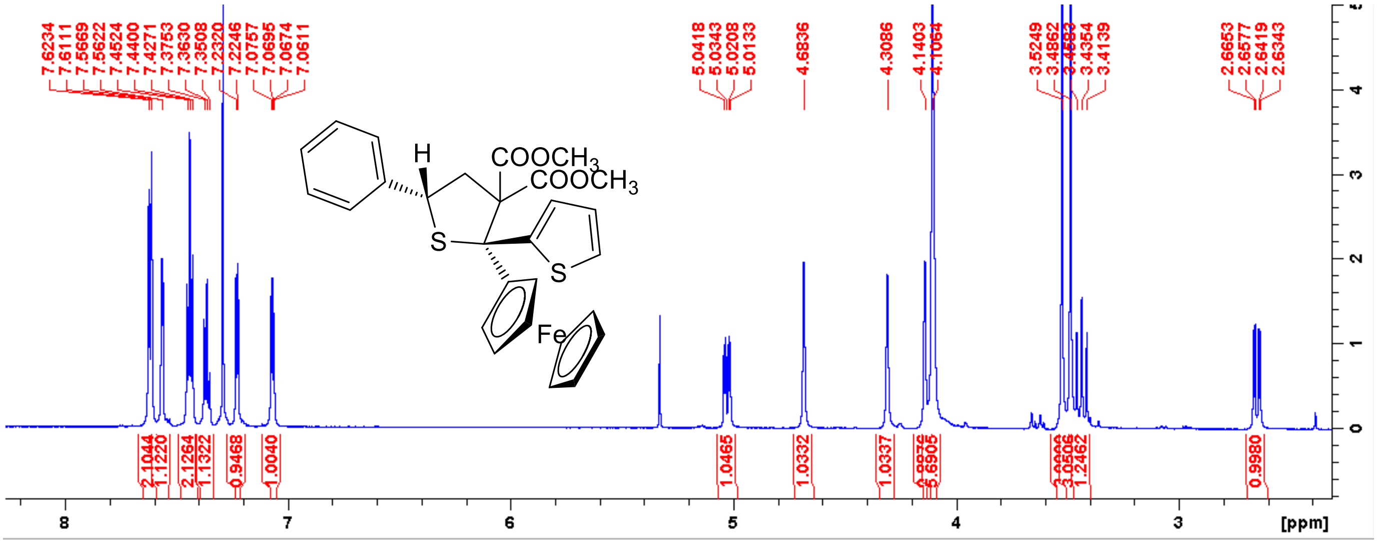

Figure S29: ^{1}H NMR spectrum for **9l**.

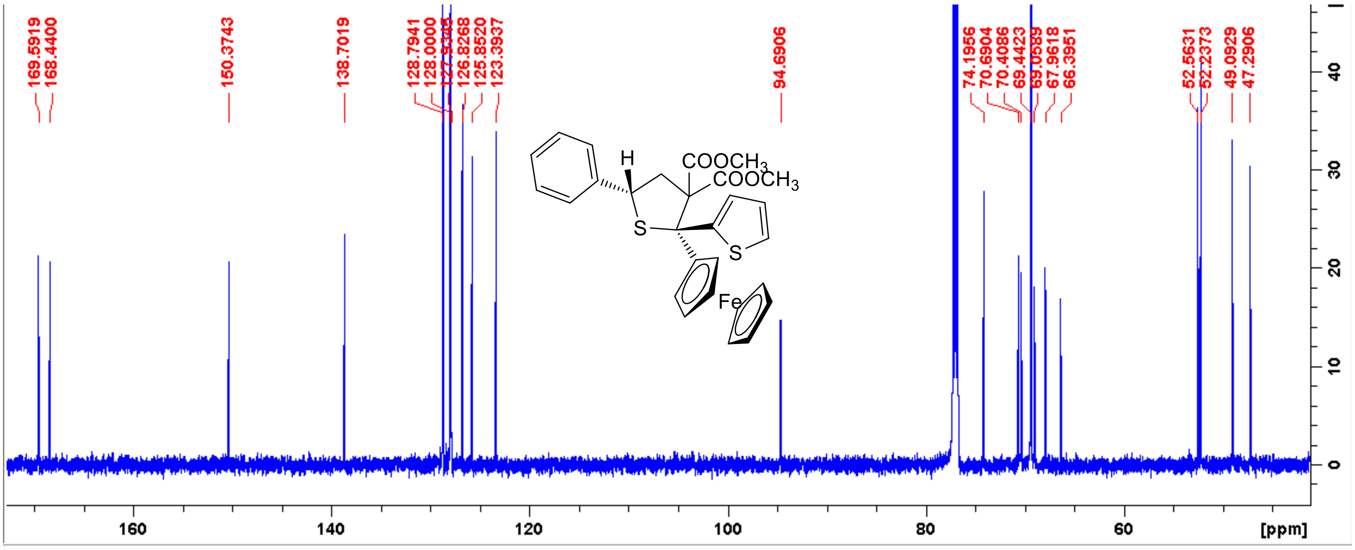

Figure S30: ^{13}C NMR spectrum for **9l**.

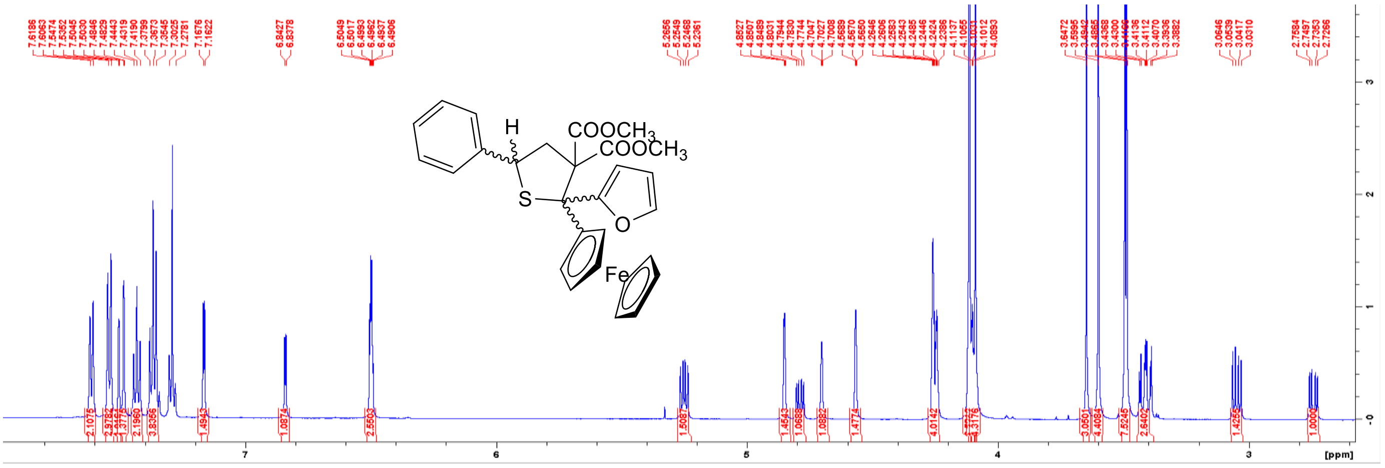

Figure S31: ^{1}H NMR spectrum for **9m**.

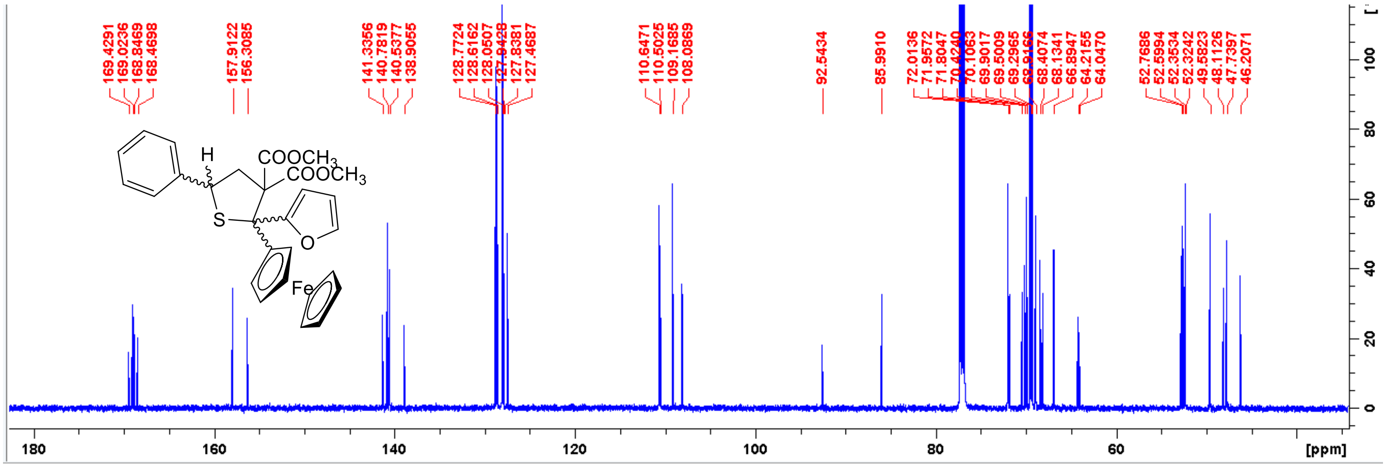

Figure S32: ^{13}C NMR spectrum for **9m**.

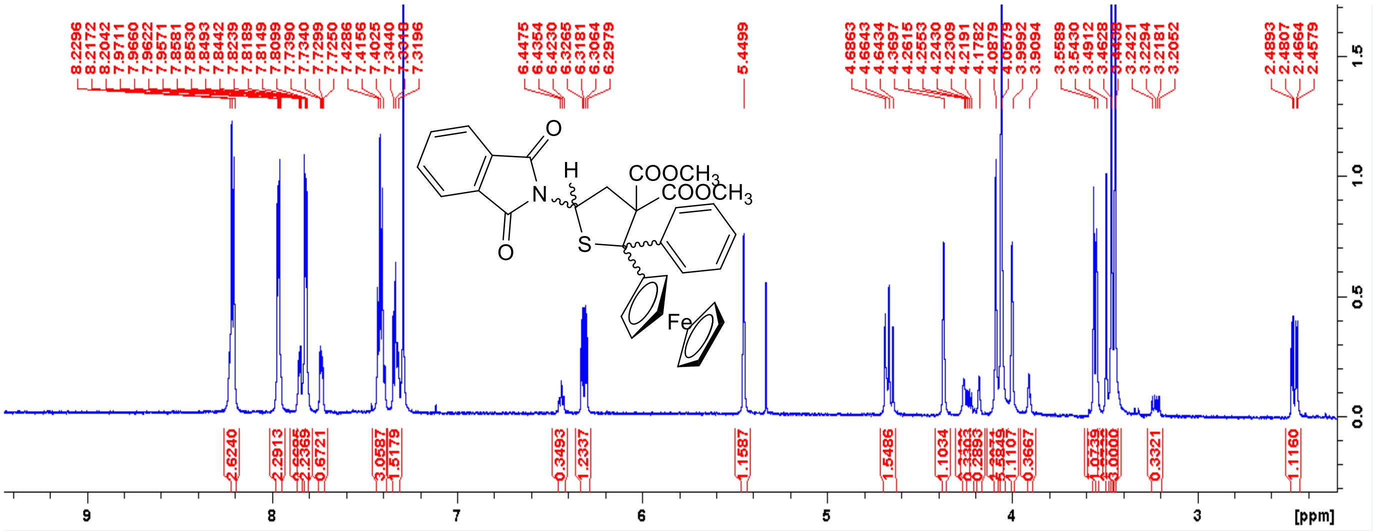

Figure S33: ^{1}H NMR spectrum for **9n**.

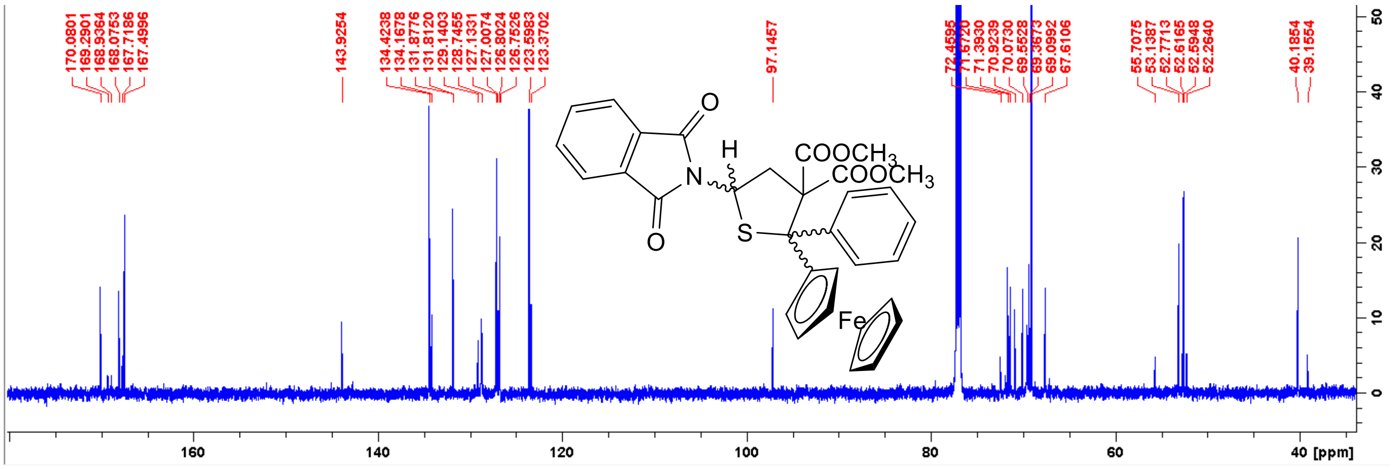

Figure S34: ^{13}C NMR spectrum for **9n**.

References

[S1] G. M. Sheldrick, *Acta Cryst*. **C71**, 3–8 (2015).
